# Urease Inhibitory Activity of *Tilia rubra* subsp. *caucasica* V.Engl.: Activity-Guided Fractionation of a Folk Medicine

**DOI:** 10.3390/molecules31213934

**Published:** 2026-11-01

**Authors:** Elif Dilmaç, İlhan Gürbüz, Gökçen Eren, Erdal Bedir

**Affiliations:** 1 Department of Pharmacognosy, Faculty of Pharmacy, Gazi University, 06330 Ankara, Türkiye; elifdilmac@gazi.edu.tr (E.D.); igurbuz@gazi.edu.tr (İ.G.); 2 Department of Pharmaceutical Chemistry, Faculty of Pharmacy, Gazi University, 06330 Ankara, Türkiye; gokcene@gazi.edu.tr; 3 Department of Bioengineering, İzmir Institute of Technology, 35430 İzmir, Türkiye

**Keywords:** *Tilia rubra*, urease inhibition, jack bean urease, flavonol glycosides, methylene-bridged dimer, guaijaverin, isoquercitrin

## Abstract

Bacterial ureases play a key role in the pathogenesis of various diseases, including gastritis, peptic ulcers, urolithiasis, and gastric cancer. Concerns regarding the toxicity and stability of urease inhibitors have prompted the search for safer alternatives from natural sources. Therefore, this study aimed to identify the bioactive compounds responsible for the urease inhibitory activity of *Tilia rubra* subsp. *caucasica* V.Engl. [synonym of *Tilia dasystyla* subsp. *caucasica* (V.Engl.) Pigott] using activity-guided fractionation. The plant material was subjected to liquid–liquid partitioning followed by chromatographic techniques, with in vitro urease inhibitory activity against jack bean urease evaluated at each step. The structures of the isolated compounds were elucidated by LC-HRMS and NMR spectroscopy. The 80% ethanol extract exhibited notable in vitro urease inhibitory activity (47.14 ± 2.59%). The ethyl acetate subextract was the most active fraction (61.38 ± 0.64% inhibition; IC_50_ = 71.35 ± 3.48 µg/mL), yielding several compounds, including guaijaverin, *trans*-tiliroside, isoquercitrin, protocatechuic acid, and a new dimeric flavonoid glycoside (methylenebisastragalin). Guaijaverin (71.86 ± 3.55% inhibition; IC_50_ = 119.63 ± 3.64 μM) and isoquercitrin (48.04 ± 2.28%) showed the strongest urease inhibitory activity at 100 μg/mL. HPLC quantification showed that guaijaverin and isoquercitrin contribute to the activity of the ethylacetate subextract; however, the compounds responsible for the observed activity could not be fully identified. Molecular docking studies revealed XP GScores of −8.64 and −8.47 kcal/mol for these compounds, respectively. These findings indicate that *T. rubra* subsp. *caucasica* is a promising natural source of urease inhibitors, warranting further experimental validation, particularly against bacterial ureases, including that of *Helicobacter pylori*, to evaluate its therapeutic potential.

## 1. Introduction

*Tilia* L. (linden), belonging to the family Malvaceae, comprises approximately 38 species and is distributed across the Northern Hemisphere, including Europe, Asia, and North America [1]. *Tilia* species are popular landscape trees owing to their shade, ornamental value, and pollution tolerance, and they also help reduce environmental pollution through heavy metal adsorption. Plants of this genus are of considerable economic value, and various parts of the plant are utilized for different purposes [2]. Their wood is used in carpentry and beekeeping; leaves are used as animal fodder; flowers are used as a nectar source for honey production and for medicinal and cosmetic purposes; and seed oil is used in soap manufacturing [2,3]. In Türkiye, various parts of *Tilia* species are widely used as folk remedies for the treatment of colds and gastrointestinal disorders, as well as for their diaphoretic, antitussive, and sedative effects [4,5,6,7,8,9,10,11,12,13]. Flavonoids, coumarins, phenolic acids, and terpenic compounds have been isolated from or detected in the flowers, bracts, leaves, and fruits of different *Tilia* species [14,15,16,17]. Furthermore, previous studies have demonstrated the anxiolytic, antidepressant [18], anti-inflammatory, antinociceptive [19], anticancer [20], antimicrobial [21], and antioxidant [22] properties of various *Tilia* species. Therefore, further phytochemical and pharmacological investigations of the *Tilia* genus are warranted to better understand its therapeutic potential and identify bioactive compounds of pharmacological interest.

The present study focused on *Tilia rubra* subsp. *caucasica* V.Engl. (TRC), currently accepted as *Tilia dasystyla* subsp. *caucasica* (V.Engl.) Pigott, one of the less-studied taxa of the genus *Tilia*. In the present study, the synonym *T. rubra* subsp. *caucasica* is retained to maintain consistency with the botanical identification and the previous literature on this taxon. This taxon is distributed in Iran, Crimea, the North Caucasus, Transcaucasia, and Türkiye [23]. In folk medicine, infusions or decoctions prepared from the plant’s flowers are used to treat colds, stomach disorders, and asthma, and as a diaphoretic, cough suppressant, and sedative in Anatolia [4,5,7,8,10,11,12].

Despite its widespread traditional use in the regions where it grows, very few studies have examined this taxon. Phytochemical investigations have revealed that methanol extracts of its flowers are rich in flavonoids, including quercetin, rhamnazin, and rutin, as well as phenolic compounds such as abscisic acid, epicatechin, and ferulic acid [24]. The available studies have also demonstrated several biological activities of the taxon, including inhibitory effects on collagenase and lipase, as well as antimicrobial and antioxidant effects. For instance, Subaş et al. (2023) reported that a methanol bark extract inhibited the enzymes collagenase and lipase with IC_50_ values of 78 and 81 μg/mL, respectively [25]. Furthermore, the ethanol extracts of flowers and leaves exhibited antimicrobial activity against various bacterial and fungal strains, with MIC values ranging from 0.5 to 8.0 mg/mL [26]. Ethanol extracts prepared from the flowers, leaves, and stems exhibited antioxidant activity in both DPPH (IC_50_: 0.106–0.231 mg/mL) and CUPRAC (0.272–0.552 mmol Trolox/g) assays, with the leaf extract showing the strongest activity [24]. Although these studies have revealed some promising biological activities, the phytochemical composition and biological properties of TRC remain insufficiently investigated. Furthermore, to the best of our knowledge, urease-inhibitory activity has not been investigated in any *Tilia* species, and isolation-based phytochemical studies on TRC inflorescences are lacking. TRC, which exhibited urease-inhibitory activity in preliminary experiments, was therefore selected for further investigation. Accordingly, the present study aimed to further characterize its phytochemical profile and to investigate its biological activity in line with its traditional use.

The urease enzyme (urea amidohydrolase, EC 3.5.1.5) increases the pH of the environment by hydrolyzing urea into ammonia and carbamate. Bacterial ureases play a role in the pathogenesis of many diseases, such as gastritis, peptic ulcers, urinary stone formation, catheter obstruction, pyelonephritis, ammonia encephalopathy, hepatic coma, and gastric cancer. *Helicobacter pylori* urease is particularly critical in this context. The bacterium counteracts low pH conditions with the help of urease; as a result of this enzyme’s activity, the microenvironmental pH is nearly neutralized, allowing the bacterium to survive in the acidic environment of the stomach. During this process, live *H. pylori* cells can safely reach the mucus layer by adsorbing enzymes released during the autolysis of other *H. pylori* cells [27]. In addition to facilitating *H. pylori* survival in the acidic gastric environment, urease contributes to pathogenesis by stimulating monocytes and neutrophils, thereby promoting the release of inflammatory cytokines and epithelial cell damage. Rising resistance of *H. pylori* to clarithromycin, metronidazole, and levofloxacin has lowered the eradication rates of standard therapies [28], which has renewed interest in non-antibiotic targets such as urease. Furthermore, urease activity has been associated with the survival of *Mycobacterium tuberculosis*. Urease also plays a significant role in urinary tract infections and urinary stone formation caused by urease-producing microorganisms, including *Proteus mirabilis*, *Ureaplasma urealyticum*, *Staphylococcus* spp., *Klebsiella* spp., and *Pseudomonas* spp. [27,29]. Beyond their therapeutic potential, urease inhibitors are environmentally important, as excessive soil urease activity following the application of urea-based fertilizers accelerates ammonia volatilization, resulting in nutrient loss, plant damage, and environmental pollution. Therefore, urease inhibitors have attracted considerable interest for both medical and agricultural applications [30].

Urease inhibitors are considered promising agents for the treatment of various diseases caused by urease-dependent pathogenic microorganisms. However, current urease inhibitors, such as phosphorodiamidates, hydroxamic acid derivatives, and imidazoles, have limitations related to toxicity and low stability, which may restrict their therapeutic application. Therefore, the discovery of new urease inhibitors with high stability and low toxicity is crucial for human health and has become the focus of numerous studies [31,32]. In this regard, the rich chemical diversity of natural sources, especially plants, offers a unique resource. Such sources may provide compounds that are difficult or uneconomical to synthesize and that have diverse mechanisms of action [33].

In this context, the urease-inhibitory activity of TRC, which is also a folk remedy, was detected, and the constituents contributing to this activity were investigated using activity-guided fractionation (AGF). Furthermore, this study contributes to the characterization of the phytochemical composition of TRC, for which limited information is currently available.

## 2. Results and Discussion

**Scheme 1:**
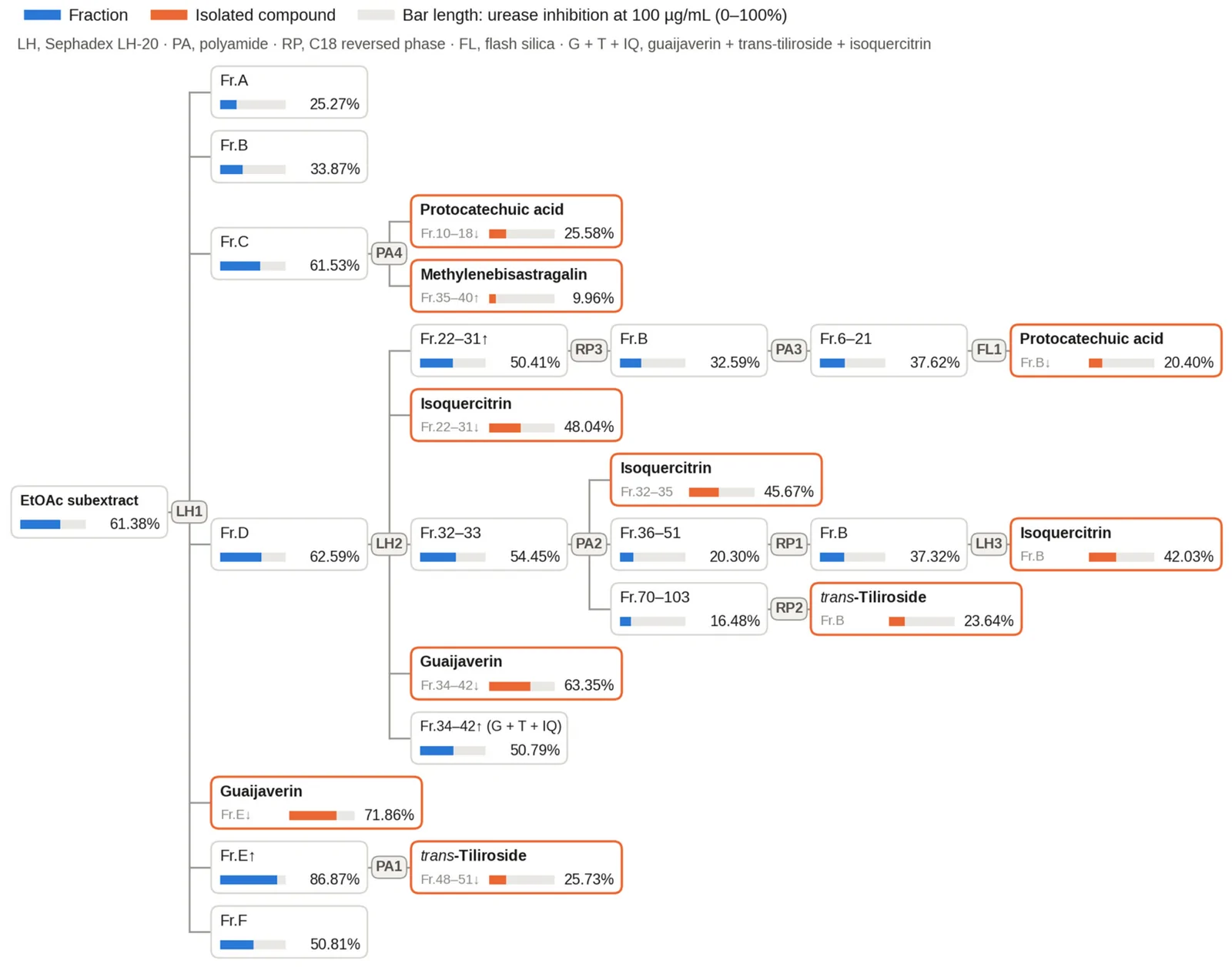
Activity-guided isolation of urease inhibitors from TRC inflorescences. Each box shows the urease inhibition at 100 µg/mL as a bar on a 0–100% scale; orange boxes denote isolated compounds. LH, Sephadex LH-20; PA, polyamide; RP, C18 reversed phase; FL, flash silica chromatography; ↑, supernatant; ↓, precipitate; G + T + IQ, guaijaverin + *trans*-tiliroside + isoquercitrin.

**Figure 1:**
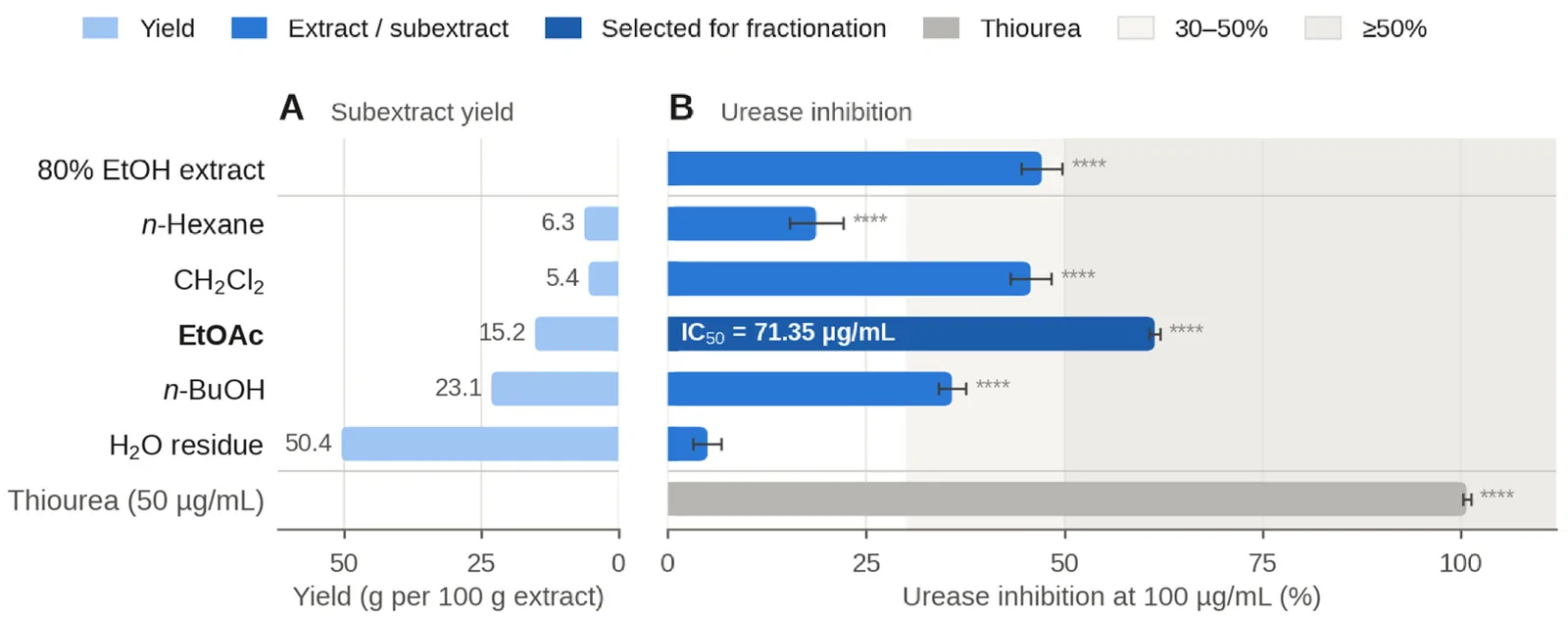
Yield calculated based on the 80% ethanol extract (**A**) and urease inhibitory activity (**B**) of the 80% ethanol extract of TRC inflorescences and its subextracts. Yields are given as g of subextract obtained from 100 g of the 80% ethanol extract. Samples were tested at 100 µg/mL and thiourea (positive control) at 50 µg/mL. Data are expressed as mean ± SD (*n* = 3, representing three technical replicates from a single experiment performed on the same plate). Shaded areas mark 30–50% and ≥50% inhibition. Asterisks denote significance versus the solvent control (80% ethanol; one-way ANOVA followed by Dunnett’s test): **** *p* < 0.0001.

**Figure 2:**
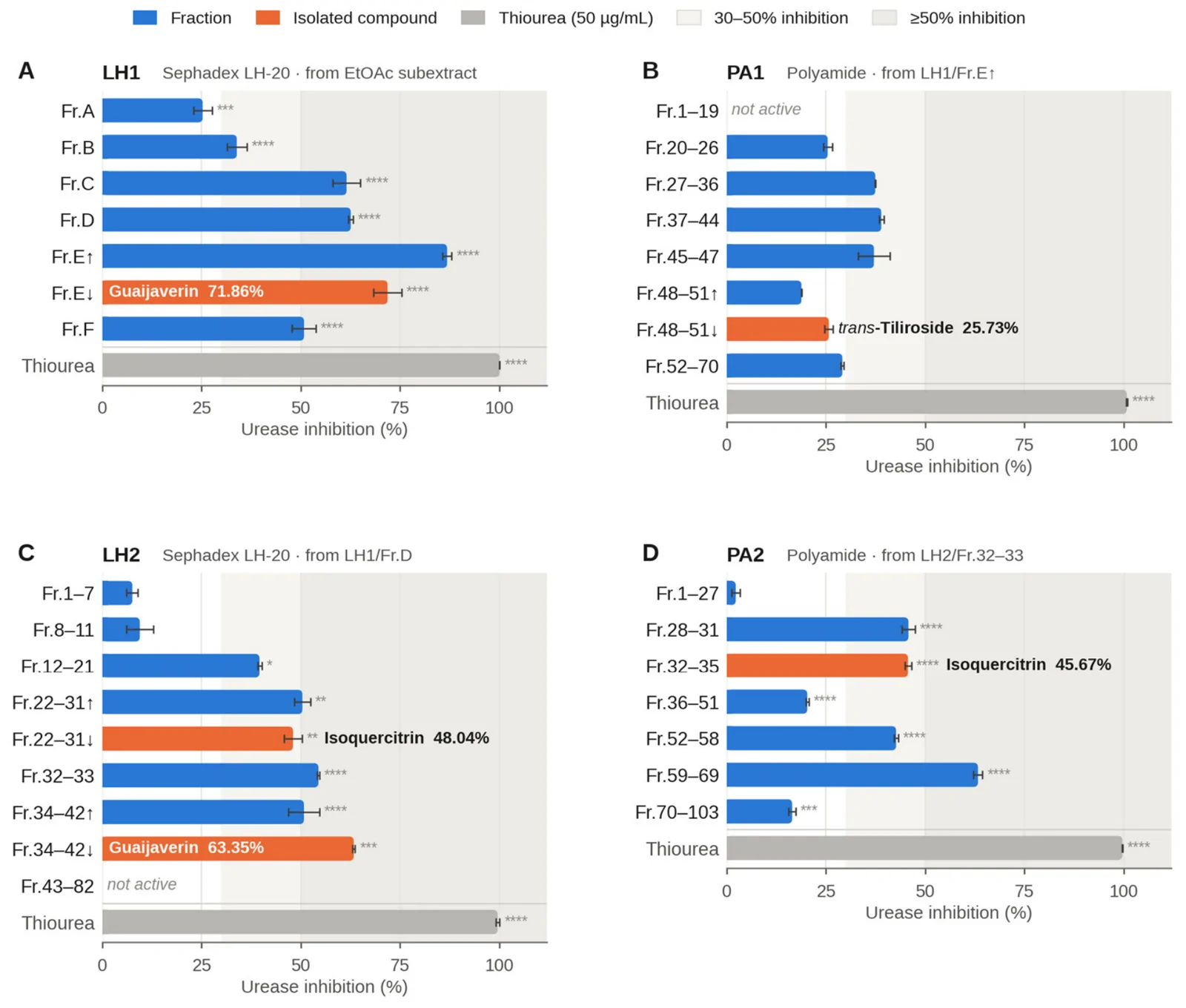
Urease inhibitory activity of the fractions obtained from the LH1 (**A**), PA1 (**B**), LH2 (**C**) and PA2 (**D**) columns; the fraction loaded onto each column is given in the panel header. Orange bars denote isolated compounds. Fractions were tested at 100 µg/mL and thiourea (positive control) at 50 µg/mL; “not active” indicates no inhibition at the tested concentration. Data are expressed as mean ± SD (*n* = 3, representing three technical replicates from a single experiment performed on the same plate). Shaded areas mark 30–50% and ≥50% inhibition. Asterisks denote significance versus the solvent control (80% ethanol; one-way ANOVA followed by Dunnett’s test): * *p* < 0.05, ** *p* < 0.01, *** *p* < 0.001, **** *p* < 0.0001.

**Figure 3:**
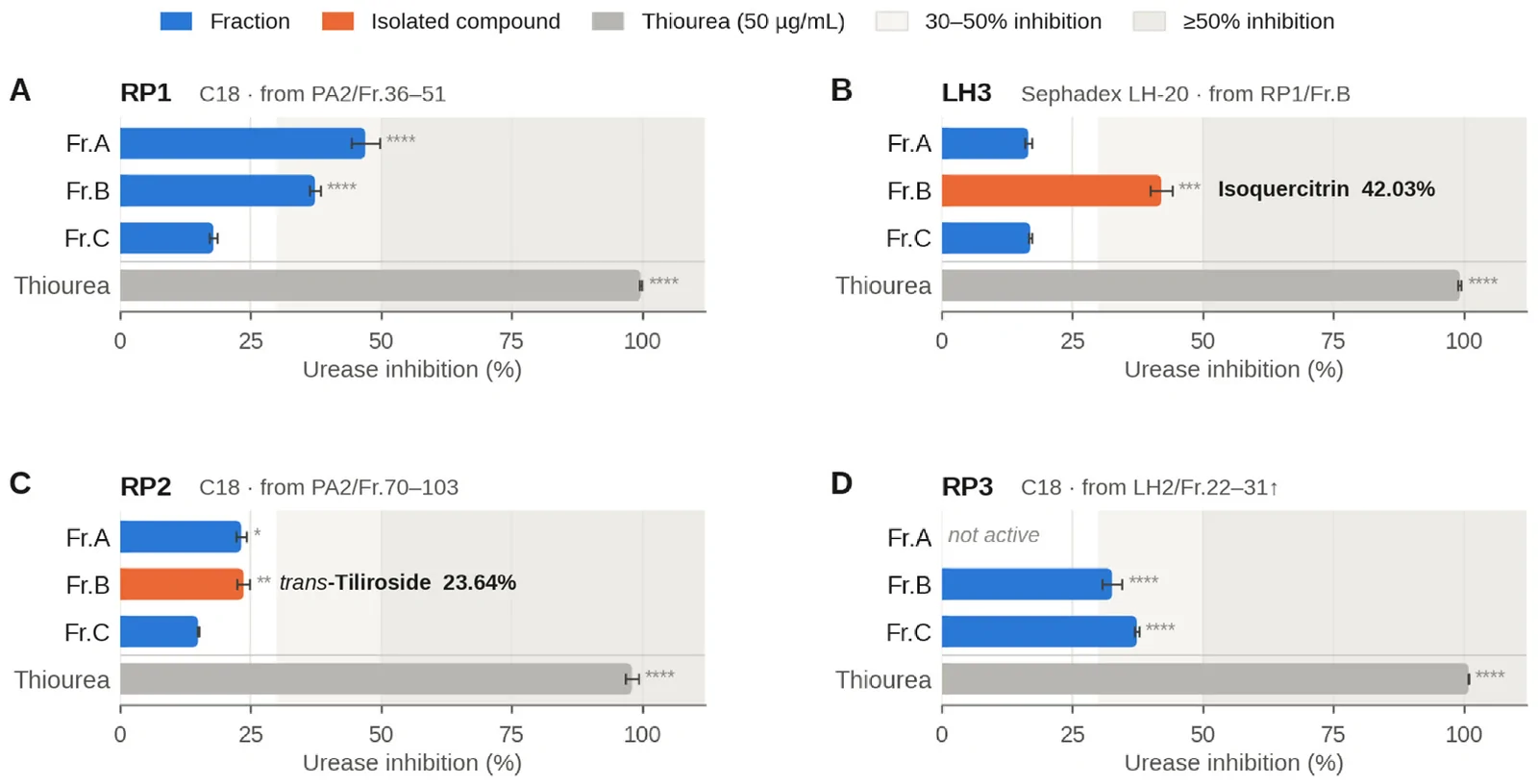
Urease inhibitory activity of the fractions obtained from the RP1 (**A**), LH3 (**B**), RP2 (**C**) and RP3 (**D**) columns; the fraction loaded onto each column is given in the panel header. Orange bars denote isolated compounds. Fractions were tested at 100 µg/mL and thiourea (positive control) at 50 µg/mL; “not active”, no inhibition at the tested concentration. Data are expressed as mean ± SD (*n* = 3, representing three technical replicates from a single experiment performed on the same plate). Shaded areas mark 30–50% and ≥50% inhibition. Asterisks denote significance versus the solvent control (80% ethanol; one-way ANOVA followed by Dunnett’s test): * *p* < 0.05, ** *p* < 0.01, *** *p* < 0.001, **** *p* < 0.0001.

**Figure 4:**
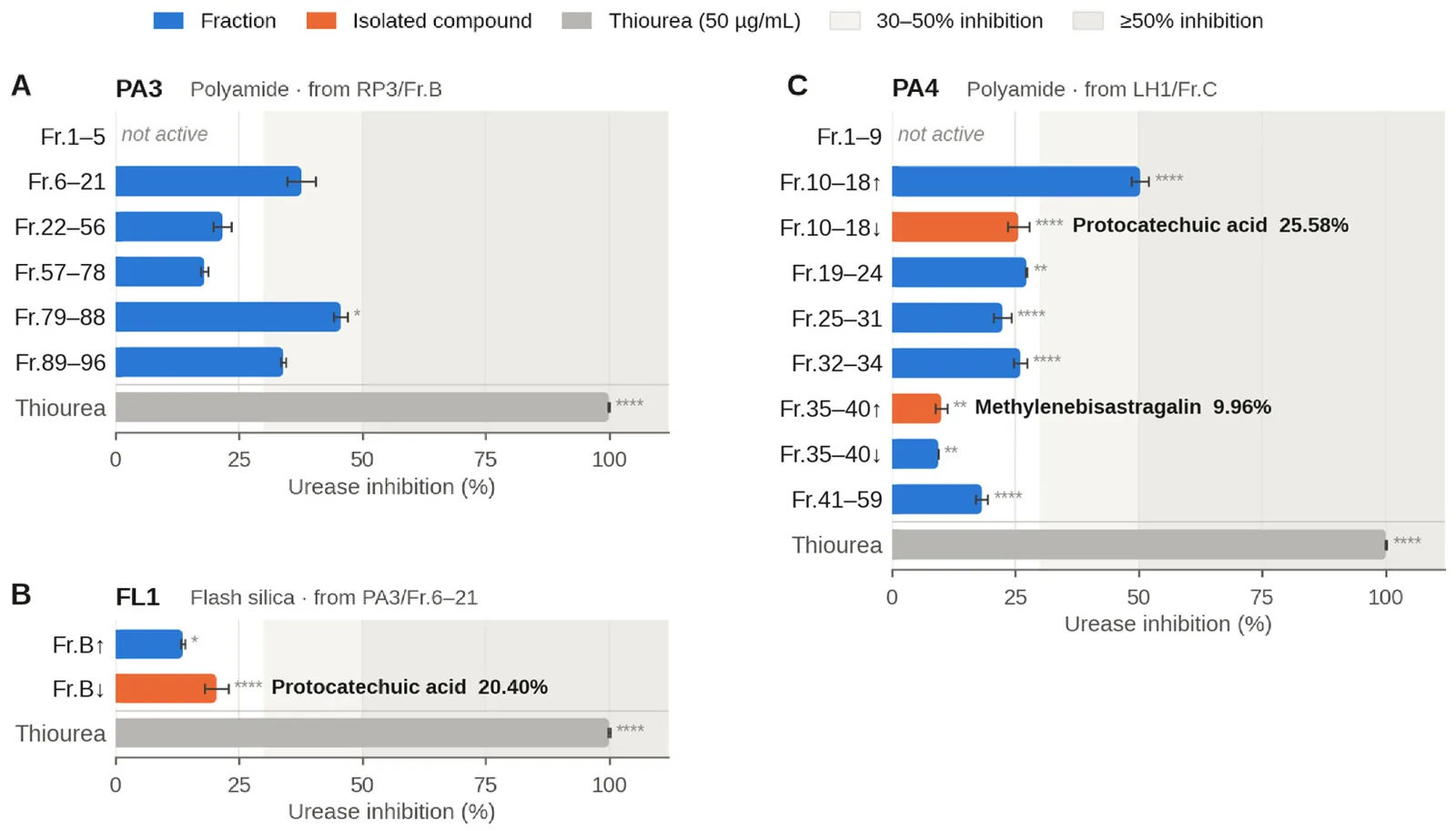
Urease inhibitory activity of the fractions obtained from the PA3 (**A**), FL1 (**B**) and PA4 (**C**) columns; the fraction loaded onto each column is given in the panel header. Orange bars denote isolated compounds. Fractions were tested at 100 µg/mL and thiourea (positive control) at 50 µg/mL; “not active” indicates no inhibition at the tested concentration. Data are expressed as mean ± SD (*n* = 3, representing three technical replicates from a single experiment performed on the same plate). Shaded areas mark 30–50% and ≥50% inhibition. Asterisks denote significance versus the solvent control (80% ethanol; one-way ANOVA followed by Dunnett’s test): * *p* < 0.05, ** *p* < 0.01, **** *p* < 0.0001.

The inflorescences of TRC, which are traditionally used for various gastric disorders [4,5,8,10,11], were investigated for their in vitro urease inhibitory activity. The urease inhibitory assay was performed using jack bean urease (*Canavalia ensiformis*), a widely accepted model enzyme for the preliminary screening of urease inhibitors due to its commercial availability and conserved active-site architecture [34]. An 80% ethanol extract of TRC was prepared and found to exhibit 47.14% (*p* < 0.0001) urease-inhibitory activity. Subsequently, the ethanol extract was subjected to liquid–liquid partitioning, and the activities of the resulting subextracts (*n*-hexane, dichloromethane, ethyl acetate, *n*-butanol, and the remaining water) were evaluated as well. As shown in Figure 1, the ethyl acetate subextract showed the strongest activity with an inhibition rate of 61.38 ± 0.64% (*p* < 0.0001) and an IC_50_ value of 71.35 ± 3.48 µg/mL (values are expressed as mean ± standard deviation, SD). Based on these results, activity-guided fractionation (AGF) was carried out on the ethyl acetate fraction of TRC. Thiourea, used as the reference inhibitor, showed an IC_50_ value of 7.46 ± 0.06 µg/mL (97.95 ± 0.51 µM), with a 95% confidence interval (CI) of 5.649–9.872 µg/mL (74.21–129.69 µM). The activity-guided fractionation procedure used in this study is summarized in Scheme 1, and the in vitro urease inhibition values of the obtained samples are presented in Figure 1, Figure 2, Figure 3 and Figure 4.

Based on the TLC profile of the ethyl acetate subextract, fractionation was initiated using a Sephadex LH-20 column (LH1). Precipitation was observed in fraction LH1/Fr.E obtained from this column. The precipitate and supernatant were designated as LH1/Fr.E↓ and LH1/Fr.E↑, respectively. After purification, the precipitate (LH1/Fr.E↓) was obtained as 48.3 mg with an HPLC purity of 96.14% and exhibited 71.86% urease-inhibitory activity at a concentration of 230 µM (*p* < 0.0001), with an IC_50_ of 51.93 μg/mL (119.63 µM; 95% CI: 72.68–208.20 µM). (The guaijaverin/thiourea IC_50_ ratio was 3.18). Subsequent structural elucidation studies identified the purified compound as guaijaverin, indicating that it is one of the compounds contributing to the urease-inhibitory activity of the ethyl acetate subextract. Among the fractions obtained from the column, the highest activity was exhibited by fraction LH1/Fr.E↑, with an inhibition rate of 86.87% and an IC_50_ value of 32.28 ± 0.46 µg/mL, followed by LH1/Fr.D, with an inhibition rate of 62.59% (*p* < 0.0001) and an IC_50_ value of 98.93 ± 4.37 µg/mL, and LH1/Fr.C, with an inhibition rate of 61.53% (*p* < 0.0001) and an IC_50_ value of 96.14 ± 3.25 µg/mL. In the same experimental set, thiourea, used as the reference inhibitor, showed an IC_50_ value of 2.86 ± 0.15 µg/mL (37.57 ± 1.97 µM), with a 95% CI of 2.124–4.400 µg/mL (27.90–57.80 µM). LH1/Fr.E↑ was subsequently subjected to further separation using a polyamide (PA1) column. Eight fractions were obtained, showing urease-inhibitory activities ranging from 18.81% to 38.93%. In the same experimental set, thiourea, used as the reference inhibitor, showed an IC50 value of 8.84 ± 0.09 µg/mL (116.27 ± 1.17 µM), with a 95% CI of 7.033–11.31 µg/mL (92.39–148.58 µM). The reduced activity after fractionation may be attributed to several factors, including the retention of polyphenolic constituents, particularly condensed tannins, on the polyamide column, and/or possible degradation during fractionation. In addition, following this separation, the precipitated portion PA1/Fr.48–51 was obtained as 3.4 mg with an HPLC purity of 89.75% and was found to consist of a single compound. After purification, this compound was identified as *trans*-tiliroside and was found to exhibit low urease-inhibitory activity, with an inhibition rate of 25.73% (at a concentration of 168 µM).

Isolation procedures were continued on another active fraction obtained from the LH1 column, LH1/Fr.D, using different separation techniques. TLC analysis revealed the presence of two major flavonoid compounds, along with several other secondary metabolites. To remove impurities, the fraction was rechromatographed on a Sephadex LH-20 column (LH2). From the LH2 column, precipitates were observed in fractions LH2/Fr.22–31 and LH2/Fr.34–42. After appropriate purification, LH2/Fr.22–31↓ was obtained as 9.3 mg with an HPLC purity of 89.37% and exhibited 48.04% urease inhibitory activity (*p* < 0.01) (at a concentration of 215 µM). Subsequent structural elucidation studies identified the purified compound as isoquercitrin, indicating that it contributes to the urease inhibitory activity of this fraction. Similarly, after purification, LH2/Fr.34–42↓ was obtained as 17.9 mg with an HPLC purity of 97.31% and exhibited a significant inhibition of 63.35% (*p* < 0.001) (at a concentration of 230 µM) with an IC_50_ value of 65.31 ± 1.85 µg/mL (150.45 ± 4.26 µM; 95% CI: 111.50–209.39 µM). Subsequent studies identified the purified compound as guaijaverin. In the same experimental set, thiourea, used as the reference inhibitor, showed an IC_50_ value of 9.19 ± 0.33 µg/mL (120.88 ± 4.41 µM; 95% CI: 7.268–11.76 µg/mL, 95.48–154.49 µM). The guaijaverin/thiourea IC_50_ ratio was 1.24, providing a reference for comparisons across different experimental conditions.

The relatively high activity observed in fractions LH2/Fr.32–33 (54.45% inhibition, *p* < 0.0001; IC_50_ = 90.62 ± 4.14 µg/mL) and LH2/Fr.22–31↑ (50.41% inhibition, *p* < 0.01; IC_50_ = 92.38 ± 2.03 µg/mL) compared with the other fractions obtained from the column indicated that activity-guided fractionation (AGF) should primarily be continued on these fractions. Evaluation of the TLC profile of LH2/Fr.32–33 revealed the presence of one major flavonoid and two minor brownish-purple spots. The fraction LH2/Fr.32–33 was applied to a polyamide column (PA2), yielding seven fractions. Among these, PA2/Fr.32–35 was found to be nearly pure; therefore, it was purified by washing with various solvents, and the major compound with a flavonoid structure was obtained in pure form as 14.9 mg, with an HPLC purity of 90.09%. Its urease-inhibitory activity was determined to be 45.67% (*p* < 0.0001) (at a concentration of 230 µM), and further studies identified the compound as isoquercitrin. Thiourea showed an IC_50_ value of 7.91 ± 0.15 µg/mL (104.09 ± 1.97 µM; 95% CI: 6.268–10.33 µg/mL, 82.34–135.71 µM). Among the fractions obtained from PA2, the highest activity was observed in PA2/Fr.59–69; however, due to its low yield and the presence of four spots along with impurities in its TLC profile, further purification steps were not pursued (Figure 2). Similarly, in the active fractions PA2/Fr.28–31 and PA2/Fr.52–58, isoquercitrin was identified as the major compound. Given their low amounts, it was considered that the observed activity in these fractions was mainly attributable to isoquercitrin; thus, no further purification steps were carried out. It was also observed that PA2/Fr.36–51 contained a high proportion of isoquercitrin, along with several minor spots. Isolation studies were continued in order to obtain these minor constituents. PA2/Fr.36–51 was first subjected to an RP1 column, and among the obtained fractions, RP1/Fr.A (46.92%, *p* < 0.0001) and RP1/Fr.B (37.32%, *p* < 0.0001) showed relatively high activity. Thiourea showed an IC_50_ of 8.60 ± 0.33 µg/mL (113.12 ± 4.35 µM; 95% CI: 6.09–11.59 µg/mL, 80.01–152.26 µM). Since the amount of RP1/Fr.A was insufficient, no further purification steps were performed. The fraction RP1/Fr.B, on the other hand, was found to be nearly pure and was further purified using a Sephadex LH-20 column (LH3). After purification with LH3, fraction LH3/Fr.B was obtained. The purified fraction was obtained as 15.6 mg with an HPLC purity of 81.36% and exhibited 42.03% (*p* < 0.001) (at a concentration of 230 µM) urease-inhibitory activity (thiourea IC_50_ = 9.12 ± 0.80 µg/mL, 120.02 ± 10.49 µM; 95% CI: 6.637–11.53 µg/mL, 87.19–151.47 µM). Further studies identified the purified compound as isoquercitrin. Although its activity was relatively low, the fraction PA2/Fr.70–103, which was considered to be nearly pure, was subsequently applied to a reversed-phase C18 column (RP2) for further isolation. RP2/Fr.B, obtained as 15.6 mg with an HPLC purity of 82.72%, exhibited 23.64% urease-inhibitory activity (at a concentration of 168 µM) (*p* < 0.01) (thiourea IC_50_ = 8.93 ± 0.46 µg/mL, 117.48 ± 6.06 µM; 95% CI: 6.328–14.11 µg/mL, 83.13–185.37 µM) and was identified as *trans*-tiliroside.

Activity-guided fractionation (AGF) was also continued on the fraction LH2/Fr.22–31↑, and this fraction was initially evaluated by TLC. The TLC profile of LH2/Fr.22–31↑, which exhibited an inhibition rate of 50.41% (*p* < 0.01) (IC_50_ = 92.38 ± 2.03 µg/mL), revealed the presence of guaijaverin and isoquercitrin as major compounds, along with trace amounts of other secondary metabolites. Therefore, these compounds were considered responsible for the activity of LH2/Fr.22–31. For further purification, LH2/Fr.22–31↑ was applied to a C18 column (RP3), during which a decrease in activity was observed. Examination of the TLC profile of RP3/Fr.C indicated that, in addition to the major compounds guaijaverin and isoquercitrin, there was one minor compound and an even fainter spot. However, despite the application of various chromatographic techniques, the amounts of the fractions decreased significantly before a pure compound could be isolated, and the process was discontinued at this stage. Nevertheless, subsequent studies showed that the faint spot observed in RP3/Fr.C corresponded to protocatechuic acid (Figure 3).

TLC analysis showed that RP3/Fr.B gave two additional spots besides guaijaverin and isoquercitrin; therefore, it was subjected to a polyamide column (PA3) for further purification. Among the obtained fractions, PA3/79–88 exhibited high activity (45.59%, *p* < 0.05) (thiourea IC_50_ = 9.33 ± 0.32 µg/mL, 122.79 ± 4.28 µM; 95% CI: 6.942–12.06 µg/mL, 91.20–158.43 µM). Evaluation of its TLC profile revealed the presence of two major flavonoids, with isoquercitrin present in a greater proportion. Accordingly, the observed activity of this fraction was considered to be mainly due to isoquercitrin. On the other hand, the TLC profile of PA3/6–21 indicated the presence of two detectable compounds along with a higher level of impurities. Therefore, further purification was carried out using flash column chromatography (FL1). Precipitation was observed in FL1/Fr.B, and after purification, protocatechuic acid was obtained as 18.8 mg with an HPLC purity of 92.46%. Protocatechuic acid was also shown to be present in RP3/Fr.C, and its urease-inhibitory activity was found to be 20.40% (*p* < 0.0001) (at a concentration of 649 µM) (thiourea IC_50_ = 6.69 ± 0.22 µg/mL, 88.02 ± 2.96 µM; 95% CI: 5.045–9.319 µg/mL, 66.28–122.43 µM).

Another active fraction obtained from the LH2 column was LH2/Fr.34–42↑. TLC analysis of this fraction revealed the presence of guaijaverin, *trans*-tiliroside, and isoquercitrin, along with some impurities. Since these compounds had already been isolated, no further separation of this fraction was considered necessary. Isolation procedures were continued on another active fraction obtained from the LH1 column, LH1/Fr.C, using a polyamide column (PA4). In PA4/Fr.10–18, one major spot was observed along with impurities and several minor spots on the TLC profile. The formation of a precipitate was noted; this precipitate was carefully separated and appropriately purified, yielding 5 mg of the major compound with an HPLC purity of 85.54%. The compound was identified as protocatechuic acid and exhibited 25.58% urease-inhibitory activity (*p* < 0.01) (at a concentration of 649 µM). TLC analysis of the most active fraction obtained from the PA4 column, PA4/Fr.10–18↑, revealed that approximately 80% of the fraction consisted of protocatechuic acid, along with minor impurities and spots corresponding to five different secondary metabolites. Given the limited amount of the fraction, it was concluded that even if the unknown secondary metabolites were isolated, their quantities would be insufficient for structural elucidation and activity assays; therefore, no further purification steps were undertaken. Among the fractions obtained from PA4, all except PA4/Fr.10–18↑ exhibited very low urease inhibitory activity. Since these fractions did not demonstrate significant activity, further purification studies were not prioritized. However, given their potential contribution to the plant’s phytochemical profile, additional trials were conducted wherever possible. Evaluation of the obtained fractions indicated that their TLC profiles were not sufficiently clean and that they were not rich in secondary metabolites; therefore, no further steps were pursued. Another fraction obtained from the column, PA4/Fr.35–40, exhibited a nearly pure profile upon TLC analysis. It was observed that part of the fraction precipitated in ethyl acetate, while the remainder stayed dissolved in the ethyl acetate phase. The precipitated portion was considered to consist of impurities, whereas the ethyl acetate phase was found to consist predominantly of a nearly pure compound (PA4/Fr.35–40↑). This compound was further purified using Sephadex LH-20 columns and was obtained as 15 mg with an HPLC purity of 80.32%. TLC analysis revealed the presence of additional phenolic constituents, indicating that minor impurities remained in the isolated fraction. Based on structural elucidation studies, it was identified as a novel compound. Because the compound contains two astragalin units linked by a methylene bridge, it was named methylenebisastragalin. Its urease inhibitory activity was determined to be 9.96% (*p* < 0.01) (at a concentration of 110 µM) (thiourea IC_50_ = 6.72 ± 0.25 µg/mL, 88.37 ± 3.33 µM; 95% CI: 4.517–10.04 µg/mL, 59.34–131.90 µM) (Figure 4).

Another active fraction obtained from the LH1 column, LH1/Fr.F, exhibited significant inhibitory activity (50.81%). However, TLC analysis revealed a complex impurity profile without clearly distinguishable secondary metabolites. Further fractionation using Sephadex LH-20 and silica gel column chromatography did not yield fractions with improved TLC profiles or identifiable major secondary metabolites. Therefore, further purification of LH1/Fr.F was not pursued.

Guaijaverin; yellow powder, LC-HRMS [M+H]^+^ *m*/*z* = 435.0926 mass error= +1.0 ppm (C_20_H_18_O_11_). ^1^H NMR (500 MHz, DMSO-*d*_6_,/δ ppm, *J* Hz): δ 7.63 (*dd*, 8.5, 2.2 Hz, H-6′), 7.49 (*d*, 2.2 Hz, H-2′), 6.82 (*d*, 8.5 Hz, H-5′), 6.38 (*d*, 2.1 Hz, H-8), 6.18 (*d*, 2.1 Hz, H-6), 5.25 (*d*, 5.1 Hz, H1″), 3.74 (*t*, 6.1 Hz, H-3″), 3.64 (*dt*, 6.0, 3.0 Hz, H-4″), 3.58 (*dd*, 11.5, 5.5 Hz, H5″a), 3.50 (*dd*, 7.1, 3.3 Hz, H-2″), 3.20 (*dd*, 11.5, 2.7 Hz, H-5″b). ^13^C: δ 177.61 (C-4), 164.34 (C-7), 161.31 (C-5), 156.39 (C-2, C-9), 148.71 (C-4′), 145.08 (C-3′), 133.83 (C-3), 122.14 (C-6′), 121.00 (C-1′), 115.87 (C-2′), 115.48 (C-5′), 104.00 (C-10), 101.52 (C-1″), 98.81 (C-6), 93.66 (C-8), 71.76 (C-2″), 70.84 (C-3″), 66.21 (C-4″), 64.44 (C-5″) [35,36]. The corresponding HPLC chromatogram is provided in Figure S1.

*trans*-Tiliroside; orange-yellow powder, LC-HRMS [M+H]^+^ *m*/*z* = 595.1458 mass error= +2.0 ppm (C_30_H_26_O_13_). ^1^H NMR (600 MHz, CH_3_OH-*d*_4_/δ ppm, *J* Hz): δ 7.99 (*d*, 8.9 Hz, H-2′, H-6′), 7.40 (*d*, 15.9 Hz, H-7‴), 7.32 (*d*, 8.6 Hz, H-2‴, H-6‴), 6.83 (*d*, 8.9 Hz, H-3′, H-5′), 6.81 (*d*, 8.6 Hz, H-3‴, H-5‴), 6.32 (*d*, 2.1 Hz, H-8), 6.14 (*d*, 2.1 Hz, H-6), 6.07 (*d*, 15.9 Hz, H-8‴), 5.24 (*d*, 7.3 Hz, H-1″), 4.29 (*dd*, 11.7, 2.3 Hz, H-6″a), 4.19 (*dd*, 11.7, 6.6 Hz, H-6″b), 3.50–3.41 (*m*, H-2″, H-3″, H-5″) 3.36–3.32 (*m*, H-4″). ^13^C: δ 178.01 (C-4), 167.34 (C-9‴), 164.62 (C-7), 161.55 (C-5), 160.09 (C-4′), 159.75 (C-4‴), 157.93 (C-2), 157.02 (C-9), 145.12 (C-7‴), 133.71 (C-3), 130.89 (C-2′ or C-6′), 130.76 (C-2′ or C-6′), 129.75 (C-2‴ or C-6‴), 128.42 (C-2‴ or C-6‴), 125.66 (C-1‴), 121.36 (C-1′), 115.36 (C-3‴,C-5‴), 114.61 (C-3′, C-5′), 113.28 (C-8‴), 104.14 (C-10), 102.45 (C-1″), 98.59 (C-6), 93.39 (C-8), 76.55 (C-3″), 74.37 (C-2″), 70.29 (C-4″, C-5″), 62.82 (C-6″) [37,38]. The corresponding HPLC chromatogram is provided in Figure S2.

Isoquercitrin; yellow powder, LC-HRMS [M+H]^+^ *m*/*z* = 465.1016 mass error= −2.5 ppm (C_21_H_20_O_12_). ^1^H NMR (500 MHz, DMSO-*d*_6_,/δ ppm, *J* Hz): δ 7.55 (*m*, H-2′, H-6′), 6.82 (*d*, 9 Hz, H-5′), 6.38 (*d*, 2.1 Hz, H-8), 6.18 (*d*, 2.1 Hz, H-6), 5.43 (*d*, 7.0 Hz, H-1″), 3.56 (*d*, 11.6 Hz, H-6″a), 3.35–3.15 (*m*, H-2″, H-3″, H-6″b), 3.10–3.05 (*m*, H-4″, H-5″). ^13^C: δ 177.44 (C-4), 164.30 (C-7), 161.26 (C-5), 156.36 (C-2), 156.18 (C-9), 148.50 (C-4′), 144.85 (C-3′), 133.33 (C-3), 121.18 (C-6′), 121.63 (C-1′), 116.20 (C-5′), 115.23 (C-2′), 103.94 (C-10), 100.89 (C-1″), 98.72 (C-6), 93.55 (C-8), 77.59 (C-5″), 76.53 (C-3″), 74.12 (C-2″), 69.95 (C-4″), 60.99 (C-6″) [36,39]. The corresponding HPLC chromatogram is provided in Figure S3.

Protocatechuic acid; brownish-white powder, LC-HRMS [M-H]^−^ *m*/*z* = 153.0193 mass error= −0.2 ppm (C_7_H_6_O_4_). ^1^H NMR (600 MHz, CH_3_OH-*d*_4_/δ ppm, *J* Hz) δ 7.45 (*d*, 2.0 Hz, H-2), 7.40 (*dd*, 8.2, 2.0 Hz, H-6), 6.76 (*d*, 8.2 Hz, H-5). ^13^C: δ 149.07 (C-4), 144.70 (C-3), 122.55 (C-1, C-6), 116.98 (C-5), 114.57 (C-2) [40,41]. The carboxyl carbon signal was not observed in the ^13^C NMR spectrum, possibly due to its weak intensity under the experimental conditions. The identification of protocatechuic acid was therefore further supported by comparison with a protocatechuic acid standard using TLC and HPLC. The isolated compound showed a corresponding TLC spot with a blue-brown coloration after visualization with 5% FeCl_3_. In addition, HPLC standard addition with protocatechuic acid supported the identification. The relevant HPLC chromatograms are provided in Figure S4.

Methylenebisastragalin; [8-((5,7-dihydroxy-2-(4-hydroxyphenyl)-4-oxo-3-(((3*R*,4*S*,5*S*,6*R*)-3,4,5-trihydroxy-6-(hydroxymethyl)tetrahydro-2*H*-pyran-2-yl)oxy)-4*H*-chromen-8-yl)methyl)-5,7-dihydroxy-2-(4-hydroxyphenyl)-3-(((3*S*,4*R*,5*R*,6*S*)-3,4,5-trihydroxy-6-(hydroxymethyl)tetrahydro-2*H*-pyran-2-yl)oxy)-4*H*-chromen-4-one] (the D-configuration of the glucose units was tentatively assigned based on literature comparisons); orange-yellow powder, UV–Vis λmax ≈ 270, 328 nm (Figure S23), LC-HRMS [M+H]^+^ *m*/*z* = 909.2053 (Figure S22) mass error = −3.4 ppm. (C_43_H_40_O_22_). An additional ion at *m*/*z* 747.17 was observed, showing a mass difference of approximately 162 Da from [M+H]^+^, consistent with a possible loss of a hexose moiety. HPLC purity: 80.32% (250 nm); the corresponding chromatogram is provided in Figure S5. Upon examination of the compound’s physical properties, TLC profile (faint yellow–orange spot under daylight, a dark spot under UV_254_ and UV_365_ nm) and UV spectrum, it was hypothesised that the structure might be a flavonoid. Given the high molecular mass detected, the possibility of a dimeric flavonoid was also considered. Among the low-intensity aromatic signals in the compound’s ^1^H-NMR spectrum (Table 1, Figures S6 and S7), attributable to a flavonoid skeleton, the singlet at δ 6.21 indicated a pentasubstituted A ring. The signals at δ 7.79 (*d*, *J* = 8.5 Hz, 4H) and 6.75 (*d*, *J* = 8.7 Hz, 4H), indicating an AA’BB’ system, indicated a para-substituted B ring in the flavonoid skeleton. The intense resonances observed in the higher-field region (δ_H_ 3.2–5.2 ppm) further confirmed the presence of a glycosylated flavonoid. The isolated anomeric proton signal at δ_H_ 5.17, with a coupling constant of *J* = 7.7 Hz, indicated that the sugar moiety possessed a *β*-configuration. The remaining sugar proton resonances were observed in the δ_H_ 3.20–3.67 ppm region. The assignments of the sugar unit were established not only from the ^1^H and ^13^C NMR spectra (Table 1, Figures S8 and S9), but also with the aid of the two-dimensional COSY (Figures S10–S14) and HSQC spectra (Figures S19–S21). Similarly, the spin systems of the flavonoid skeleton and the corresponding carbon atoms were assigned. Comparison of the NMR data of the sugar unit with the literature confirmed the presence of a glucopyranosyl moiety. The absolute configuration of the glucose unit was not experimentally determined. Based on the consistent identification of D-glucose in glycosidic constituents previously reported from this genus, the glucose unit in this compound was tentatively assumed to have the D-configuration on biogenetic grounds. After all the peaks in the ^1^H-NMR spectrum had been assigned to the flavonoid skeleton and the sugar unit, an additional signal remained (δ 4.24, s, 2H). The HSQC spectrum showed that this δ 4.24 resonance corresponded to a methylene carbon observed at δ 16.25. When the NMR data presented above were evaluated, particularly considering the mass and molecular formula, it was concluded that two equivalent flavonoid monoglycoside units might be linked to each other via a methylene (CH_2_) bridge.

**Figure 5:**
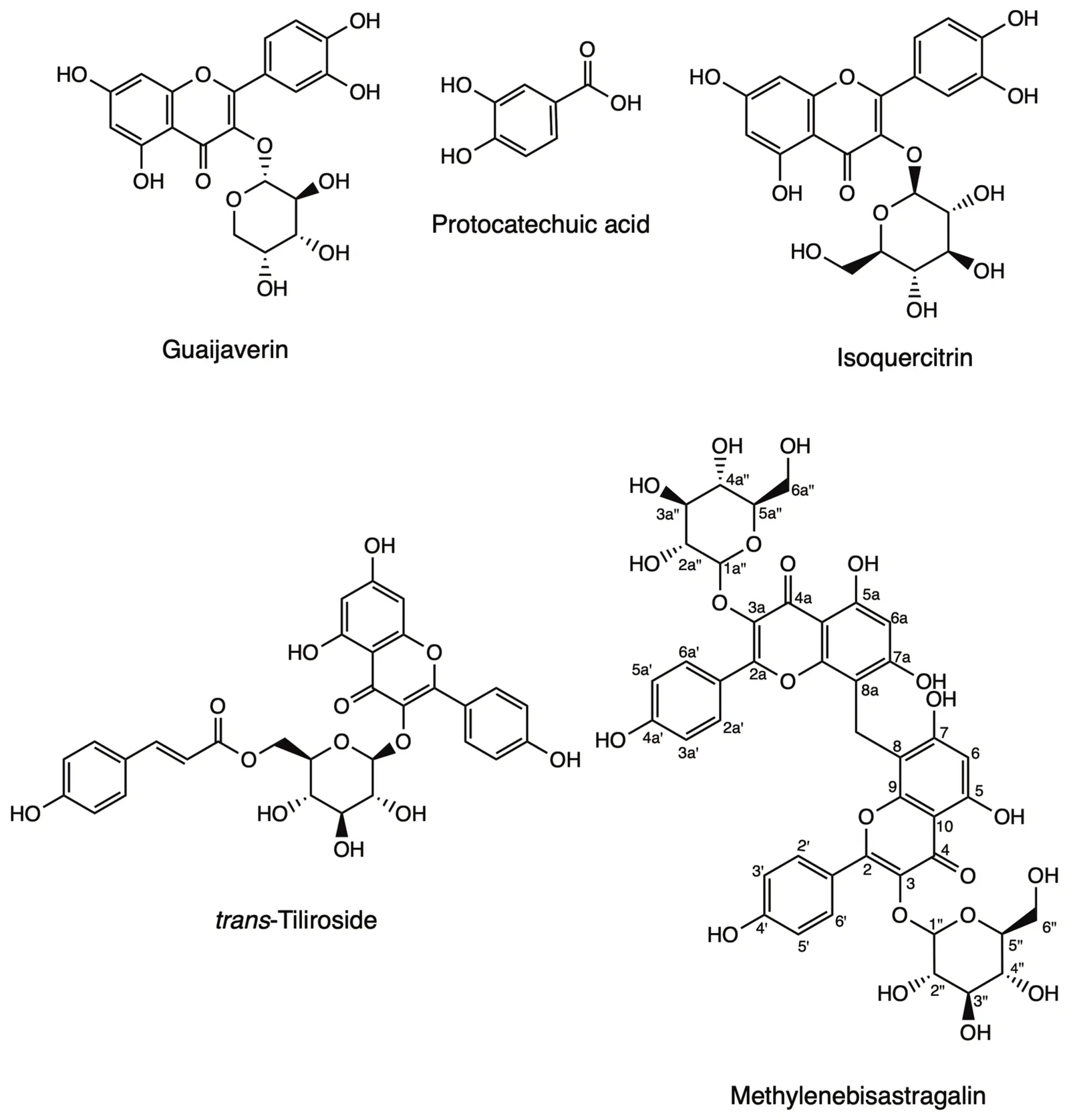
Chemical structures of the compounds isolated from TRC by activity-guided fractionation.

An HMBC spectrum (Table 1, Figures S15–S18) was obtained to confirm the assignments of the flavonoid monoglycoside skeleton and to determine the linkage positions of the substituents. First, the correlation between the δ 5.17 peak attributed to the anomeric proton and the 133.93 ppm resonance assigned to C-3 indicated that the glycosidic bond is located at C-3(O). Second, the HMBC spectrum was examined to identify the dimeric bridge point, which was determined to be part of ring A. Upon examining the long-range interactions between all the characteristic carbons assigned to ring A and the single proton (δ 6.21) in this ring, it was determined that the unsubstituted carbon is C-6 (C-5/C-7/C-8 and C-10 → H-6). Consequently, it was hypothesized that the flavonoid units bearing 3-*O*-*β*-D-glucopyranose are linked via a methylene bridge at the C-8 carbon. Indeed, the long-range interactions of the methylene protons (δ 4.24) with the C-7, C-8 and C-9 carbons in the A ring clearly confirmed the validity of this inference. To assess the novelty of methylenebisastragalin, SciFinder (CAS), Reaxys, and PubChem were searched using the exact structure, molecular formula (C_43_H_40_O_22_), monoisotopic mass, and substructure searches based on the methylene-bridged bis-kaempferol skeleton, with and without glycosylation. These searches returned no identical structure, indicating that the compound is new to the literature [42,43,44]. To further assess whether methylenebisastragalin could represent an artifact formed during the isolation process, additional LC-MS analyses were performed on both 80% ethanol and EtOAc extracts freshly prepared directly from the plant material without heat treatment, as well as on the original 80% ethanol extract and EtOAc subextract used for the isolation study. The ion corresponding to methylenebisastragalin was detected in both EtOAc samples, including the EtOAc extract freshly prepared directly from the plant material without heat treatment. In the 80% ethanol extracts, the corresponding ion was observed at very low intensity, which could potentially lead to ambiguous interpretation of its presence. This weak signal may be attributed to the low abundance of methylenebisastragalin and the greater chemical complexity of the 80% ethanol extracts. HPLC analysis of the freshly prepared EtOAc extract also revealed a peak corresponding to that of the isolated compound. These findings reduce the likelihood of its formation during subsequent fractionation and isolation procedures and suggest that methylenebisastragalin may naturally occur in the plant. Nevertheless, artifact formation cannot be completely excluded, particularly because the additional LC-MS analyses were performed using low-resolution mass spectrometry. The chromatographic and mass spectrometric data obtained from these additional analyses are provided in Figures S24–S29. The chemical structures of the isolated compounds are presented in Figure 5.

Although guaijaverin and tiliroside have previously been reported in *Tilia* species, they were isolated from TRC for the first time in this study [45,46,47,48]. Isoquercitrin has been previously identified in *T. rubra*; however, it was isolated from TRC for the first time in this study [49]. In contrast, protocatechuic acid has already been reported in TRC [24]. On the other hand, methylenebisastragalin, a dimeric flavonoid glycoside formed by the linkage of two astragalin units via a methylene bridge, was isolated from a natural source for the first time.

**Figure 6:**
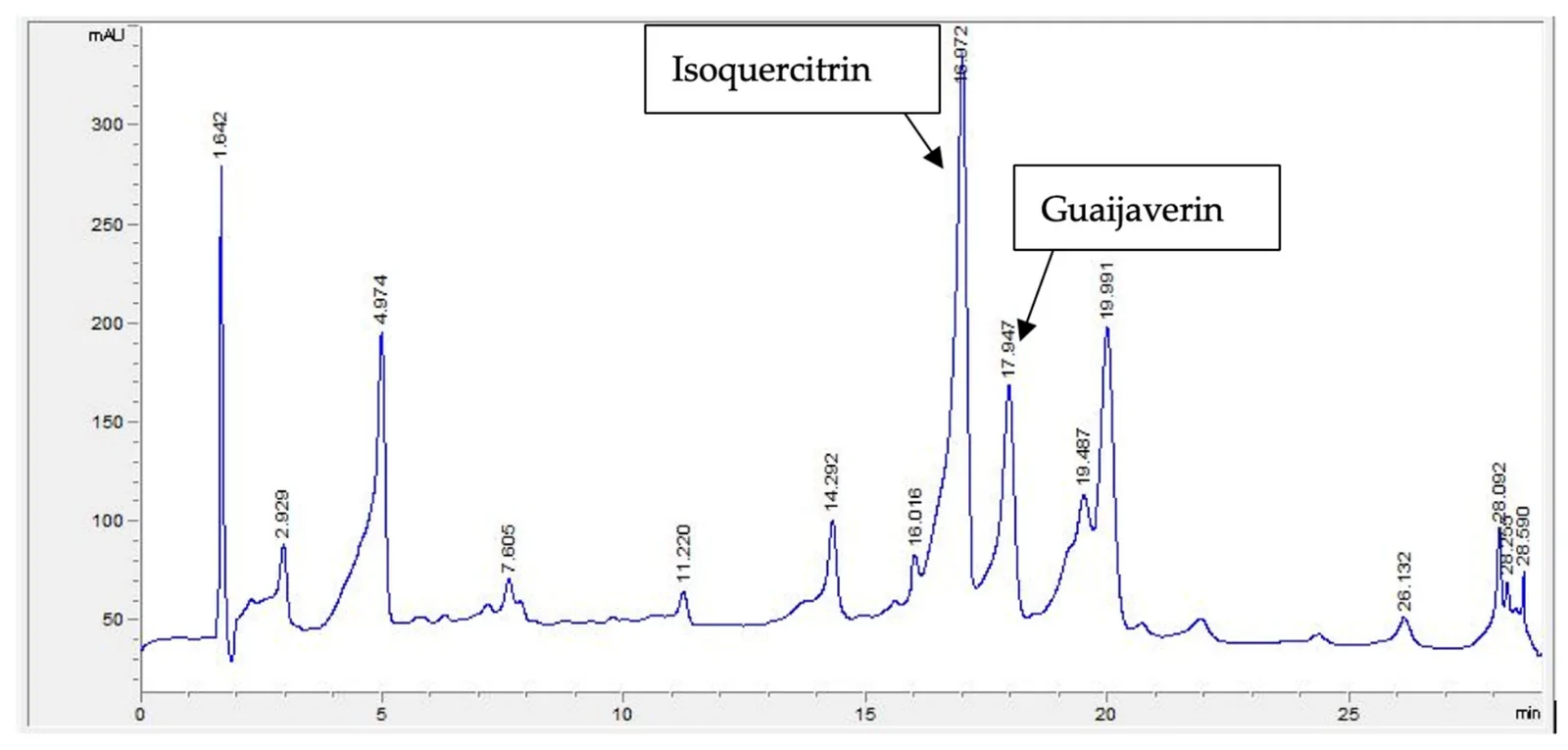
HPLC chromatogram of the ethyl acetate subextract of TRC (250 nm).

The amounts of guaijaverin and isoquercitrin isolated from the ethyl acetate subextract of TRC were determined by HPLC. The content of guaijaverin was calculated as 4.68 mg/100 mg extract, while that of isoquercitrin was 12.85 mg/100 mg extract. The chromatogram of the ethyl acetate subextract is presented in Figure 6. The retention times, LOD and LOQ values, as well as the linear relationship between peak area and concentration, are summarized in Table 2.

At 100 μg/mL, the ethyl acetate subextract contains 10.8 µM guaijaverin and 27.7 µM isoquercitrin. Considering these concentrations together with the inhibitory activities of the isolated compounds, guaijaverin and isoquercitrin may contribute to the urease inhibitory activity of the subextract but do not fully account for the observed activity. This suggests that other constituents of the subextract may also contribute to its overall inhibitory effect.

The potential binding modes and intermolecular interactions of the urease-inhibiting compounds guaijaverin and isoquercitrin within the active site of urease were investigated by molecular docking studies (Table 3). The catalytic pocket of urease consists of a deep cavity containing two nickel ions (Ni901 and Ni902), which constitute the essential catalytic center responsible for substrate hydrolysis. Direct or indirect interactions between ligands and these metal ions are considered critical determinants of urease inhibitory activity [50]. The flexible loop structure located above the catalytic pocket is referred to as the flap region. This mobile structural element functions as a gate that regulates substrate access to the active site and facilitates product release following catalysis. The flap region is primarily composed of the residues CYS592 (modeled as CME592 in the X-ray crystal structure, PDB: 4H9M) and HIS593. In addition, ARG439, ALA440, GLN635, MET637 and GLY638 line the entrance of the active site. These residues play essential roles both in shaping the active site architecture and in ensuring the proper orientation of the substrate during catalysis. Consequently, compounds capable of forming hydrogen bonds or hydrophobic interactions with flap region residues may restrict flap mobility, thereby interfering with the catalytic process and ultimately exerting urease inhibitory activity [51].

**Figure 7:**
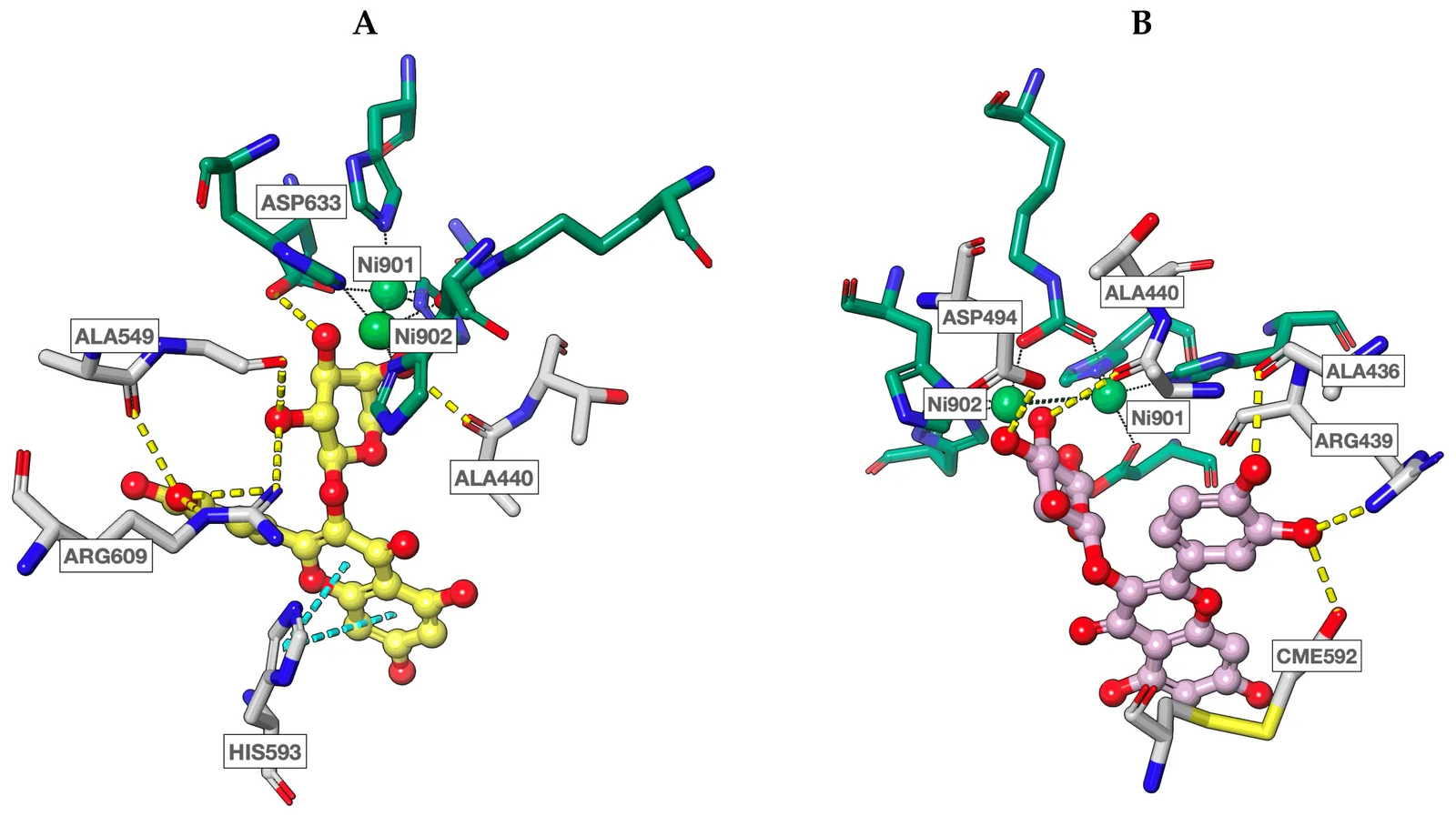
Predicted binding modes of guaijaverin (**A**) and isoquercitrin (**B**) within the active site of urease (PDB: 4H9M). Hydrogen bonds and π-π interactions are shown as yellow and cyan dashed lines, respectively.

Analysis of the docked urease-guaijaverin complex revealed that the hydroxyl groups of guaijaverin formed hydrogen bonds with the residues ALA440, ALA549, ARG609, and ASP633. In addition, the benzopyran ring engaged in π-π interactions with HIS593. Notably, the hydroxyl group was oriented toward both catalytic nickel ions (Ni901 and Ni902), suggesting possible interactions with the metal center. The XP GScore for the urease-guaijaverin complex was calculated to be −8.64 kcal/mol (Figure 7A).

According to the docking conformation obtained for isoquercitrin within the urease active site, the compound was positioned in close proximity to both catalytic nickel ions and engaged in direct coordination through its oxygen atoms. In addition, isoquercitrin formed multiple hydrogen bonds with the residues ALA436, ARG439, ALA440, ASP494, and CME592, which corresponds to a β-mercaptoethanol adduct of CYS592 (S,S-(2-Hydroxyethyl)thiocysteine) in the X-ray crystal structure. The predicted interactions suggested that isoquercitrin may establish contacts within the enzyme’s active site region. The XP GScore was calculated to be −8.47 kcal/mol (Figure 7B).

Overall, the docking results suggested that both compounds could interact with the catalytic nickel ions and surrounding residues within the urease active site, providing complementary structural insights into the potential binding modes.

In a study conducted by Shabana et al. using the indophenol method, guaijaverin isolated from *Psidium guajava* L. was reported to inhibit *C. ensiformis* urease with an IC_50_ value of 120 µM, while it showed 17% inhibition against *Lactobacillus fermentum* urease at a concentration of 100 µM. In the same study, isoquercitrin exhibited an IC_50_ value of 160 µM against *C. ensiformis* urease. In our study, guaijaverin was isolated from two different fractions with purities of 96.14% and 97.31%, exhibiting IC_50_ values of 119.63 µM and 150.45 µM, respectively. The differences in the observed inhibitory activities between the two fractions may reflect inter-assay variability rather than differences in purity, as indicated by the variation in the IC_50_ values of the reference inhibitor thiourea (2.86 and 9.19 µg/mL) between the corresponding assay sets. These findings are consistent with those reported by Shabana et al. (2010). Additionally, the well-known urease inhibitor acetohydroxamic acid exhibited markedly stronger activity, with an IC_50_ value of 20 µM against *C. ensiformis* urease and 70% inhibition against *L. fermentum* urease at 100 µM [52].

Shoukry et al. (2023), in their study on *Jacaranda mimosifolia* D. Don using the indophenol method, reported that isoquercitrin inhibited *C. ensiformis* urease with an IC_50_ of 9.10 ± 0.75 µM, whereas the standard inhibitor, thiourea, showed an IC_50_ of 3.48 ± 0.67 µM [53]. Similarly, isoquercitrin isolated from *Monotheca buxifolia* (Falc.) A. DC. demonstrated 98.6% inhibition at 0.5 mM, with an IC_50_ value of 51.6 ± 1.46 µM (thiourea IC_50_ = 21 ± 0.11 µM) [54]. In contrast, another study using the indophenol method, which expressed activity as µg thiourea equivalents/mg, reported that isoquercitrin was inactive [55]. These findings indicate considerable variability in the reported urease-inhibitory potency of isoquercitrin, ranging from IC_50_ values of 9.10 µM [53] and 51.6 µM [54] to 160 µM [52], while another study reported no activity [55]. In our study, isoquercitrin exhibited approximately 48% inhibition at 100 µg/mL, suggesting that our findings are more closely aligned with those of Shabana et al. (2010) [52] than with studies reporting substantially stronger inhibitory activity. Furthermore, a previous molecular docking study showed that isoquercitrin binds to the active site of *C. ensiformis* urease (PDB: 4GY7), forming hydrogen bonds with HIS593, ALA440, GLY550, GLN635, MET588, and ARG439, as well as π-π stacking and π-alkyl interactions with HIS593, ALA440, ALA636, and MET637. Isoquercitrin exhibited a binding constant of 0.62 µM, a binding affinity of −8.60 kcal/mol, and a MolDock score of −121.42 [56].

A review of the literature on tiliroside, another compound isolated during our activity-guided fractionation (AGF) studies, revealed that tiliroside isolated from *Oliveria decumbens* Vent. inhibited *C. ensiformis* urease with an IC_50_ value of 0.45 ± 0.05 mM, while the standard inhibitor hydroxyurea showed an IC_50_ value of 0.78 ± 0.01 mM. In the same study, molecular docking analysis revealed that tiliroside exhibited a binding affinity of −8.49 kcal/mol toward *C. ensiformis* urease (PDB: 4H9M). According to the predicted binding mode, the hydroxyl groups of the kaempferol moiety were involved in binding within the active site by coordinating with the catalytic nickel ions and forming hydrogen bonds with ASP633 and the side chain of CME592. In addition, the two hydroxyl groups of the glucoside moiety formed hydrogen bonds with the side chain of ARG439, further stabilizing the ligand-enzyme complex [57]. This study did not specify the geometric configuration (*cis/trans* or *E/Z)* of tiliroside, which limits direct comparison with the *trans*-tiliroside identified in the present study. In our study, *trans*-tiliroside showed weak urease-inhibitory activity, with inhibition values of 25.73% and 23.64% (*p* < 0.01) in different fractions. However, since the inhibition did not exceed 50% at the tested concentration, the IC_50_ value could not be determined. Although the geometric configuration (*cis/trans* or *E/Z*) of tiliroside was not specified in the literature, both the published data and our findings indicate that it exhibits limited urease-inhibitory activity.

Protocatechuic acid, another compound isolated from the inflorescences of TRC and previously reported in this species [24], has been evaluated in several studies for its urease-inhibitory and antibacterial activities against *H. pylori*. In a study conducted by Yener et al. (2020) using the indophenol method, protocatechuic acid was found to be inactive when its activity was expressed as µg thiourea equivalents per mg [55]. In contrast, Hassan and Švajdlenka (2017), using electrospray ionization–mass spectrometry, reported that protocatechuic acid isolated from *Hibiscus sabdariffa* L. inhibited urease with an IC_50_ value of 1.7 ± 0.1 µM, while acetohydroxamic acid showed lower activity (IC_50_ = 3.2 µM) [58]. In another urease inhibition study conducted on *H. pylori* isolates, protocatechuic acid exhibited up to 40% inhibition at the highest concentration tested (48 mg/L) [59]. Although Yener et al. (2020), using the same indophenol assay as in the present study, reported no urease-inhibitory activity for protocatechuic acid, the literature suggests that the observed activity may vary depending on the method employed [55]. Moreover, the weak urease inhibitory activity observed in the present study, with inhibition values of 25.58% and 20.40%, is consistent with several previous reports. Molecular docking studies conducted by Hassan and Švajdlenka further demonstrated the favorable binding of protocatechuic acid to *H. pylori* urease (PDB: 1E9Y) and *C. ensiformis* urease (PDB: 3LA4), with binding affinities of −5.9 and −5.8 kcal/mol, respectively. In the active site of *H. pylori* urease, protocatechuic acid formed hydrogen bonds with LYS445 and VAL473, along with a π-donor hydrogen bond interaction involving TYR475. Similarly, in *C. ensiformis* urease, protocatechuic acid established hydrogen-bonding interactions with GLU718, ASP730, and LYS716, indicating stable ligand-protein binding in both urease enzymes [58].

Methylenebisastragalin, a novel natural product identified in the present study, belongs to the rare class of methylene-bridged dimeric flavonoids, for which only a limited number of naturally occurring analogs have been reported to date. Previous studies have demonstrated diverse biological activities for compounds of this structural class, including antioxidant, anti-inflammatory, antimicrobial, immunomodulatory, antimalarial, cardioprotective, and anticancer effects [43,60,61,62]. To the best of our knowledge, methylenebisastragalin represents the first dimeric flavonoid reported from the genus *Tilia*. However, methylenebisastragalin was essentially inactive in the urease inhibition assay (9.96% inhibition at 100 μg/mL).

According to our literature survey, no previous studies have investigated the urease inhibitory activity of *Tilia* species. Although *Tilia* species have traditionally been used in the management of gastric disorders, scientific studies investigating this traditional use are still lacking. Therefore, the present study provides the first evaluation of the urease inhibitory activity of *T. rubra* subsp. *caucasica.* In the activity-guided fractionation, the ethanol extract of TRC inflorescences, and particularly its ethyl acetate subextract, showed urease-inhibitory activity. Among the isolated compounds, guaijaverin and isoquercitrin showed the highest inhibition percentages under the tested conditions (63.35% and 71.86% at 230 µM, and 48.04%, 45.67%, and 42.03% at 215 µM, respectively), whereas *trans*-tiliroside and protocatechuic acid (25.73% and 23.64% at 168 µM, and 20.40% and 25.58% at 649 µM, respectively) were weakly active, and methylenebisastragalin was inactive (9.96% at 110 µM). However, differences in molar concentrations should be taken into account when evaluating the relative inhibitory activities of these compounds. At their concentrations in the subextract, guaijaverin and isoquercitrin explain only part of its activity, indicating that other constituents may also contribute to the observed inhibitory effect. Therefore, the compounds responsible for the overall urease inhibitory activity of the subextract could not be fully identified. As discussed above, the available literature is partly consistent with the urease inhibitory activities observed for these compounds. In addition, tannins and proanthocyanidins may contribute to the observed urease inhibitory activity through nonspecific interactions with proteins [32,63]. Their potential redistribution or removal during fractionation may partly explain the reduction in inhibitory activity observed in some fractions. Regarding the isolated compounds, according to our literature survey, methylenebisastragalin, a novel natural product identified in the present study, is the first methylene-bridged dimeric flavonoid reported from the genus *Tilia*. However, due to the limited amounts of the isolated compounds, their combined urease inhibitory activity could not be evaluated. Collectively, these findings expand the limited phytochemical and pharmacological knowledge of TRC and highlight this species as a promising subject for further investigation of its urease-inhibitory constituents.

## 3. Materials and Methods

### 3.1. Materials

*n*-Butanol (98.0%, general purpose reagent, Lab-Scan, Dublin, Ireland), dichloromethane (>99.0%, GC, Ph. Eur., NF, Sigma-Aldrich, St. Louis, MO, USA), ethyl acetate (99.0%, general purpose reagent, Lab-Scan, Ireland), *n*-hexane (95%, general purpose reagent, Lab-Scan, Ireland), methanol (≥99.9%, LiChrosolv^®^, Reag. Ph. Eur., Merck, Darmstadt, Germany), sulfuric acid (92.95%, Birpa, Ankara, Türkiye), C18 reversed-phase silica gel (Fluka, Buchs, Switzerland; Merck LiChroprep, Germany), thin-layer chromatography plates (TLC Silica Gel 60 F254, Merck, Germany), PTFE syringe filters 45/25 mm (Isolab, Eschau, Germany), Sephadex LH-20 (Sigma LH20100, Fine Chemicals, Pharmacia, Stockholm, Sweden), silica gel (Merck, Germany), disodium hydrogen phosphate (Ph. Eur., BP, 98.5–101%, Sigma-Aldrich), ethylenediaminetetraacetic acid (EDTA) (anhydrous, crystalline, BioReagent, suitable for cell culture, Sigma-Aldrich, USA), phenol (ACS Reagent, Ph. Eur., GR for analysis, Merck, Germany), jack bean urease (Type-III powder, 15,000–50,000 units/g solid, Sigma-Aldrich, USA), lithium chloride (ACS reagent ≥99%, Sigma-Aldrich, USA), polyamide G (Fluka, Switzerland), potassium phosphate dibasic (≥99.0%, Sigma-Aldrich), sodium dihydrogen phosphate (Emprove^®^ expert, Ph. Eur., BP, USP, Merck, Germany), sodium hydroxide (Emsure, for analysis, Merck, Germany), sodium hypochlorite (6–14% active chlorine, Tekkim, Bursa, Türkiye), sodium nitroprusside dihydrate (purum p.a. ≥98.0%, Fluka, St. Louis, MO, USA), thiourea (Reagent Plus^®^ ≥99.0%, Sigma-Aldrich, USA), urea (ACS, Reag. Ph. Eur., Merck, Germany).

### 3.2. Plant Material

Inflorescences of TRC were collected in the first week of July 2020, during the plant’s flowering period, from Büyükköy, Çayeli, Rize (350 m, 41°00′50.8″ N; 40°40′22.9″ E). The inflorescences were dried in the shade at room temperature and stored under appropriate conditions. A voucher specimen was deposited in the Gazi University Faculty of Pharmacy Herbarium under the code GUEF 3821. The material was identified by Prof. Dr. Hayri Duman (Gazi University Faculty of Science, Department of Biology).

### 3.3. Preparation of Extracts and Fractionation

The dried inflorescences (540 g) were coarsely powdered using a herb grinder and macerated with 3000 mL of 80% ethanol. Eighty percent ethanol was selected as a hydroalcoholic solvent suitable for extracting a broad range of plant constituents, as its effectiveness in the recovery of various phytoconstituents has been previously reported [64]. The maceration was carried out in a 35 °C water bath for 48 h. The extract was then filtered and concentrated in a rotary evaporator at a temperature not exceeding 40 °C. The plant residue was subjected to the same process nine more times. The extracts were combined after being filtered and concentrated in the rotary evaporator each time. Finally, the combined extract was concentrated again on a rotary evaporator and kept in a desiccator to yield 102.51 g of 80% ethanol extract (18.98% yield). Subsequently, the 80% ethanol extract (100 g) was suspended in 1 L of 90% methanol and partitioned with n-hexane (40 × 300 mL) in a separatory funnel. After the *n*-hexane extraction was complete, the methanol phase was evaporated, and the residue was suspended in 1 L of distilled water. This was sequentially extracted with dichloromethane (28 × 300 mL), ethyl acetate (52 × 300 mL), and *n*-butanol (25 × 300 mL). Each fraction was evaporated to dryness under the previous conditions, and the aqueous residue was freeze-dried. As a result, subextracts of *n*-hexane (6.26 g, 1.19% yield), dichloromethane (5.44 g, 1.03% yield), ethyl acetate (15.19 g, 2.88% yield), *n*-butanol (23.13 g, 4.39% yield), and the remaining water (50.41 g, 9.57% yield) were obtained (yields expressed relative to the dry plant material). The slight difference (0.43%) between the total mass of the subextracts and the initial extract mass may be attributable to minor moisture uptake despite storage in a desiccator and/or minor variability associated with weighing.

### 3.4. Activity-Guided Fractionation and Isolation of Active Compounds

The ethyl acetate subextract (5 g), which exhibited the highest activity, was applied to a Sephadex LH-20 column (100 g, 4 × 60 cm, flow rate 4 mL/min) and eluted with methanol; this column was coded as LH1. Fifty-five fractions were collected, and these were combined into six subfractions: LH1/Fr.A (31.1 mg), LH1/Fr.B (346.2 mg), LH1/Fr.C (1455.9 mg), LH1/Fr.D (1689.6 mg), LH1/Fr.E (338.7 mg), LH1/Fr.F (973.3 mg). Precipitation was observed in LH1/Fr.E, which was separated into LH1/Fr.E↓ and LH1/Fr.E↑. It was determined that LH1/Fr.E↓ was obtained as a purified fraction (=guaijaverin). The urease inhibitory activity of each subfraction was evaluated in vitro.

The fraction with the highest activity (LH1/Fr.E↑) was applied to a polyamide column (6.250 g, 1.5 × 19 cm) and eluted with an H_2_O:methanol gradient (100:0→0:100, flow rate 3 mL/min). Seventy fractions were collected from this column, coded as PA1, and these were combined into seven subfractions: PA1/Fr.1–19, PA1/Fr.20–26, PA1/Fr.27–36, PA1/Fr.37–44, PA1/Fr.45–47, PA1/Fr.48–51, PA1/Fr.52–70. Precipitation was observed in PA1/Fr.48–51, which was separated into PA1/Fr.48–51↓ (=*trans*-tiliroside) and PA1/Fr.48–51↑. In vitro urease inhibition experiments were performed on each column subfraction. The main active constituent(s) of LH1/Fr.E↑, the most active fraction (86.87%), could not be identified because most of its activity was lost following fractionation on the polyamide column.

Another active fraction of the LH1 column, LH1/Fr.D, was again applied to the Sephadex column under the same conditions, and the resulting 82 fractions were combined into 7 subfractions: LH2/Fr.1–7, LH2/Fr.8–11, LH2/Fr.12–21, LH2/Fr.22–31, LH2/Fr.32–33, LH2/Fr.34–42, LH2/Fr.43–82. Precipitates formed in LH2/Fr.22–31 and LH2/Fr.34–42; these fractions were separated into lower and upper phases, yielding the subfractions LH2/Fr.22–31↓ (=isoquercitrin), LH2/Fr.22-31↑, LH2/Fr.34-42↓ (=guaijaverin), LH2/Fr.34–42↑ (=guaijaverin + *trans*-tiliroside + isoquercitrin). Subsequently, the in vitro urease inhibitory activities of the nine subfractions were determined. The active subfraction obtained from the LH2 column, LH2/Fr.32–33, was applied to a polyamide column, designated PA2 (6.250 g, 1.5 × 19 cm, H_2_O:methanol gradient 100:0→0:100). The 103 fractions obtained from this column were pooled into 7 subfractions: PA2/Fr.1–27, PA2/Fr.28–31, PA2/Fr.32–35, PA2/Fr.36–51, PA2/Fr.52–58, PA2/Fr.59–69, PA2/Fr.70–103. Subfraction PA2/Fr.32–35 was purified to yield isoquercitrin. PA2/Fr.36–51 was subjected to a C18 column, designated RP1 (4 g, 1.5 × 22 cm, H_2_O:methanol gradient 85:15→0:100), and the 55 fractions obtained were combined into 3 subfractions, RP1/Fr.A, RP1/Fr.B, RP1/Fr.C, and their urease inhibitory activities were determined. Isolation was continued with RP1/Fr.B, which was applied to a Sephadex column, designated LH3. Twenty-three fractions were collected from the LH3 column (3 g, 2 × 21 cm), and these fractions were grouped into three subfractions, LH3/Fr.A, LH3/Fr.B, LH3/Fr.C, and their activities were determined. Subfraction LH3/Fr.B was purified to yield isoquercitrin. PA2/Fr.70–103 was applied to a C18 column (RP2) (14 g, 2.5 × 39 cm) and eluted with an H_2_O:methanol gradient (80:20→0:100, flow rate 0.3 mL/min). The 53 fractions were collected and grouped as RP2/Fr.A, RP2/Fr.B, and RP2/Fr.C. RP2/Fr.B was determined to be pure and was identified as *trans*-tiliroside. Another active fraction of the LH2 column, LH2/Fr.22–31↑, was applied to a C18 column (RP3) (75 g, 4 × 40 cm) and eluted with a H_2_O:methanol system (55:45 → 0:100). A total of 14 fractions were collected and grouped into three fractions (RP3/Fr.A, RP3/Fr.B, and RP3/Fr.C). Further fractionation was carried out on RP3/Fr.B, which was applied to a polyamide column (PA3) (20 g, 2.5 × 37 cm). Elution was performed using an H_2_O:methanol system (100:0 → 0:100), yielding 96 fractions, which were combined into six groups: PA3/Fr.1–5, PA3/Fr.6–21, PA3/Fr.22–56, PA3/Fr.57–78, PA3/Fr.79–88, and PA3/Fr.89–96. Fraction PA3/Fr.6–21, identified as nearly pure, was further subjected to isolation using flash column chromatography. PA3/Fr.6–21 (80 mg) was applied to a RediSep silica column (4 g) and eluted with a dichloromethane:methanol system (100:0 → 0:100). A total of 128 fractions were collected and combined into Fr.A and Fr.B. Fr.B showed precipitation and was separated into FL1/Fr.B↓ (=protocatechuic acid) and FL1/Fr.B↑.

Another active fraction of the LH1 column, LH1/Fr.C, was applied to a polyamide column (PA4) (60 g, 4 × 40 cm). Elution was performed using an H_2_O:methanol system (100:0 → 0:100), yielding 96 fractions, which were combined into seven groups (PA4/Fr.1–9, PA4/Fr.10–18, PA4/Fr.19–24, PA4/Fr.25–31, PA4/Fr.32–34, PA4/Fr.35–40, and PA4/Fr.41–59). Precipitation was observed in PA4/Fr.10–18 when kept in a methanol:water mixture; the precipitate (PA4/Fr.10–18↓) was determined to be of high purity and identified as protocatechuic acid. PA4/Fr.35–40 was precipitated at low temperature using ethyl acetate, and this process was repeated twice. TLC analysis showed that the supernatant phases were almost pure. The resulting PA4/Fr.35–40↑ was further purified using Sephadex LH-20 columns of different sizes (300–1500 mg) as needed, and the resulting fractions were combined according to their purity determined by TLC. The high purity of the final fraction was confirmed by HPLC, and it was named methylenebisastragalin.

### 3.5. Structure Elucidation of Purified Compounds

A Hewlett-Packard–Agilent 1260 LC system (HPLC) was used to assess the purity and obtain the UV spectra of the isolated compounds. An ACE 5 C18 column (5 μm, 150 × 4.6 mm) was employed, and detection was carried out using a DAD detector (210–365 nm). The system was operated at a column temperature of 25 °C and a flow rate of 1 mL/min. The analysis was performed using a methanol:water (0.1% formic acid) solvent system, starting from 20:80 to 100:0 over 20 min, followed by 2 min of methanol, giving a total run time of 22 min.

To determine the molecular weights and assess the purity of the isolated compounds, high-resolution mass spectra (HRMS) were obtained from their methanolic solutions using electrospray ionization in both positive (ESI^+^) and negative (ESI^−^) modes. Analyses were performed on a Waters LCT Premier XE liquid chromatography–time-of-flight mass spectrometer (UPLC/MS-TOF) system, and data were processed using MassLynx 4.1 software.

The ^1^H-NMR spectra of the isolated compounds LH1/Fr.E↓ (=guaijaverin) and LH2/22–31↓ (=isoquercitrin), as well as all spectra of PA4/35–40↑ (=methylenebisastragalin), were recorded using a Bruker Avance NEO 500 instrument. The ^13^C-NMR spectra of guaijaverin and isoquercitrin, and the ^1^H-NMR and ^13^C-NMR spectra of PA1/48–51↓ (=*trans*-tiliroside) and PA4/10–18↓ (=protocatechuic acid) were obtained using an Agilent Premium Compact 600 MHz (magnet: 14.1 Tesla) NMR spectrometer. Tetramethylsilane (TMS) was used as the internal standard in all spectra. Structures of the compounds are illustrated in Figure 5.

### 3.6. Quantitation of Isolated Compounds with HPLC

The amounts of guaijaverin and isoquercitrin in the ethyl acetate subextract of TRC were determined by HPLC analysis. The standard compounds used for quantification had purities of 97% and 90%. Standard solutions (5, 10, 50, 100, 250, 500 ppm) were prepared in methanol, and calibration curves were constructed by plotting peak areas against concentrations. Detection was carried out at 250 nm. The ethyl acetate subextract was prepared at a concentration of 1 mg/mL in methanol. All samples and standards were filtered through 0.45 µm membrane filters prior to analysis. Each sample was injected in triplicate (*n* = 3), except for the 5 ppm standard solution, which was injected six times (*n* = 6) to evaluate precision. The method was partially validated according to ICH Q2 guidelines, and validation parameters including r^2^, RSD% LOD, and LOQ were calculated [65].

HPLC analysis was carried out using an Agilent 1220 Infinity system equipped with a C18 column (150 × 4.6 mm, 5 µm). The mobile phase consisted of water–formic acid (100:0.5, *v*/*v*) (A) and methanol (B). The detailed HPLC gradient programme, including changes in mobile-phase composition and flow rate throughout the analysis, is presented in Table 4. The injection volume was 20 µL.

### 3.7. Urease Inhibition Assay

Urease activity was determined by measuring ammonia production using the indophenol method. First, 25 µL of jack bean urease enzyme solution (10 U/mL, prepared in Na_2_HPO_4_/NaH_2_PO_4_ phosphate buffer; pH 6.8) and 5 µL of the test sample (prepared in 80% ethanol; final ethanol concentration: 2% *v*/*v*; final sample concentration: 100 µg/mL) were added to microplate wells and incubated at 30 °C for 15 min. Subsequently, 55 µL of the substrate solution (100 mM urea, 0.01 M K_2_HPO_4_, 1 mM EDTA, and 0.01 M LiCl; pH 8.2; final urea concentration: 27.5 mM) was added to each well, followed by a second incubation at 30 °C for 15 min. After incubation, 45 µL of phenol reagent (1% *w*/*v* phenol and 0.005% *w*/*v* sodium nitroprusside) and 70 µL of alkaline reagent (0.4% *w*/*v* NaOH and NaOCl containing 0.05% active chlorine) were added to each well, resulting in a final assay volume of 200 µL. The mixture was then incubated at room temperature for 50 min, and the increase in absorbance was measured at 630 nm [66,67]. In the control and reference experiments, 80% ethanol and thiourea were used instead of the test sample, respectively. Sample-specific blanks were included at each extract concentration to correct for background absorbance. These controls accounted for sample-related absorbance, although potential interactions of polyphenols with the Berthelot reaction were not specifically evaluated using ammonium-spike recovery experiments. All experiments were performed in triplicate as technical replicates on the same plate. For statistical analysis using Dunnett’s multiple comparisons test, the solvent control (80% ethanol) was used as the reference group.

### 3.8. Molecular Docking

Molecular docking studies were carried out using the Induced Fit Docking (IFD) protocol [68,69] implemented in the Schrödinger Small-Molecule Drug Discovery Suite (Small-Molecule Drug Discovery Suite, 2024-3, Schrödinger, LLC, New York, NY, USA, 2024). The X-ray crystal structure of urease from *Canavalia ensiformis* (PDB: 4H9M) was retrieved from the RCSB Protein Data Bank and was prepared using the Protein Preparation Wizard tool. During the preparation process, all water molecules were removed, bond orders were assigned, hydrogen atoms were added, and protonation states were generated at physiological pH (7.4 ± 1.0) using Epik. No metal-binding states were explicitly assigned during ligand preparation, and no metal-coordination constraints were imposed during IFD calculations. The docking protocol was validated by redocking the co-crystallized acetohydroxamic acid ligand from the urease crystal structure (PDB: 4H9M) using the same IFD workflow. The redocked pose reproduced the experimentally observed binding mode with an RMSD of 0.8917 Å. IFD calculations were then performed. During the initial docking stage, the van der Waals (vdW) radius scaling factor was set to 0.50, and the top 20 poses for each compound were retained. Protein residues located within 5.0 Å of the ligand poses were refined using Prime to account for local conformational changes in the binding site. Subsequently, the compounds were re-docked with Glide in Extra Precision (XP) mode on the low-energy refined structures. Default Schrödinger IFD parameters were employed unless otherwise specified. The resulting docking poses were ranked according to their XP GlideScore values. The top-ranked binding conformations were selected based on docking scores and visual inspection of key protein-ligand interactions.

### 3.9. Statistical Evaluation

The IC_50_ values were calculated using GraphPad Prism 6 software. The results are expressed as mean ± standard deviation (SD). Statistical differences between the reference group and the sample groups were evaluated using one-way ANOVA based on technical replicates obtained within the same experiment. Dunnett’s multiple comparisons test was used for post hoc analysis. The degree of significance was set at *p* < 0.05 (* *p* < 0.05; ** *p* < 0.01; *** *p* < 0.001; **** *p* < 0.0001).

## 4. Conclusions

The 80% ethanol extract of TRC inflorescences and its ethyl acetate subextract exhibited urease inhibitory activity, to which guaijaverin and isoquercitrin were found to contribute, suggesting their potential for further investigation in the context of urease-related diseases. However, the present findings are based on a cell-free assay using jack bean urease, and further studies using bacterial ureases and urease-producing microorganisms would be valuable to establish the biological relevance of the observed activity. Further in vitro and in vivo studies are warranted to investigate the antibacterial effects of its urease-inhibiting subextracts and isolated active compounds against urease-producing pathogens, such as *H. pylori.* These studies should also examine their potential prophylactic or therapeutic effects on peptic ulcers, as well as their toxicological profiles. Overall, these findings expand the current knowledge of the phytochemical composition and biological activities of TRC and highlight its potential for further pharmacological investigation.

## Figures and Tables

**Table 1:** The ^1^H-NMR and ^13^C-NMR spectroscopic data of methylenebisastragalin (CH_3_OH-*d*_4_, ^1^H-NMR: 500 MHz, ^13^C-NMR: 125 MHz).

C/H		δ_C_ (ppm)	δ_H_ (ppm)/*J* (Hz)	HMBC (H→C)
**2, 2a**	C	157.96	-	
**3, 3a**	C	133.93	-	
**4, 4a**	C=O	178.26	-	
**5, 5a**	C	159.73	-	
**6, 6a**	CH	98.79	6.21 (s, 2H)	C-5,5a, C-7,7a, C-8,8a, C-10,10a
**7, 7a**	C	163.93	-	
**8, 8a**	C	105.29	-	
**9, 9a**	C	154.67	-	
**10, 10a**	C	103.99	-	
**1′, 1a′**	C	121.57	-	
**2′, 2a′**	CH	130.83	7.79 (*d*, *J* = 8.6 Hz, 4H)	C-2′,2a′/C-6′,6a′, C-4′,4a′
**3′, 3a′**	CH	114.49	6.75(*d*, *J* = 8.6 Hz, 4H)	C-1′,1a′, C-3′,3a′/C-5′,5a′, C-4′,4a′
**4′, 4a′**	C	159.45	-	
**5′, 5a′**	CH	114.49	6.75 (*d*, *J* = 8.6 Hz, 4H)	C-1′,1a′, C-3′,3a′/C-5′,5a′, C-4′,4a′
**6′, 6a′**	CH	130.83	7.79 (*d*, *J* = 8.6 Hz, 4H)	C-2′,2a′/C-6′,6a′, C-4′,4a′
**-CH_2_-**		16.25	4.24 (*s*, 2H)	C-7,7a, C-8,8a, C-9,9a
***β*** -D-Glucopyranoside *				
**1″, 1a″**	CH	103.03	5.17 (*d*, *J* = 7.7 Hz, 2H)	C-3,3a
**2″, 2a″**	CH	74.35	3.49–3.36 (*m*, 4H)*	C-1″,1a″, C-3″,3a″
**3″, 3a″**	CH	76.63	3.49–3.36 (*m*, 4H)*	C-2″,2a″ C-4″,4a″
**4″, 4a″**	CH	69.85	3.30–3.35 (2H)	C-3″,3a″, C-5″,5a″
**5″, 5a″**	CH	76.87	3.20 (*ddd*, *J* = 9.8, 5.3, 2.4 Hz, 2H).	C-4″,4a″
**6″a, 6a″a** **6″b, 6a″b**	CH_2_	61.16	3.67 (*dd*, *J* = 11.9, 2.4 Hz, 2H), 3.53 (*dd*, *J* = 11.9, 5.3 Hz, 2H)	C-4″,4a″ C-5″,5a″

* The D-configuration of the glucose units was tentatively assigned based on literature comparisons and has not been experimentally confirmed.

**Table 2:** Calibration equations, retention times, r^2^ values, precision (RSD%) values, LOD values, and the amounts of guaijaverin and isoquercitrin in the ethyl acetate subextract.

Compound	Retention Time (min)	Calibration Equation	r^2^	Precision (%RSD)	LOD (μg/mL)	LOQ (μg/mL)	Content in TRC/EtOAc Extract
Guaijaverin	17.947	y = 88.193x + 319.81	0.9994	3.71	0.346	1.050	4.68 mg/100 mg extract
Isoquercitrin	16.972	y = 49.732x + 136.86	0.9997	2.31	0.881	2.671	12.85 mg/100 mg extract

**Table 3:** Interactions of guaijaverin and isoquercitrin within the enzyme active site according to molecular docking results.

Compound	Metal Contacts	Hydrogen Bonding	Other Interactions	XP GScore (kcal/mol)
Guaijaverin	Ni901 Ni902	ALA440, ALA549, ARG609, ASP633	HIS593 (π-π)	−8.64
Isoquercitrin	Ni901 Ni902	ALA436, ARG439, ALA440, ASP494, CME592	-	−8.47

**Table 4:** HPLC gradient programme, mobile-phase composition, and flow rate.

Time (min)	Water: Formic Acid (100:0.5, *v*/*v*)	Methanol	Flow Rate mL/min
0.00	80.00	20.00	1.20
10.00	60.00	40.00	1.00
13.00	50.00	50.00	1.00
17.00	50.00	50.00	0.60
23.00	50.00	50.00	0.60
25.00	50.00	50.00	0.70
25.01	50.00	50.00	1.20
27.00	0.00	100.00	1.20
29.00	0.00	100.00	1.20

## Data Availability

The NMR spectroscopic data of methylenebisastragalin were deposited in the Natural Products Magnetic Resonance Database (NP-MRD) under the accession number NP0354539. All other datasets generated and/or analyzed during the current study are available from the corresponding author upon reasonable request.

## Supplementary Materials

The following supporting information can be downloaded at: https://www.mdpi.com/article/10.3390/molecules31213934/s1. The Supplementary Materials include HPLC chromatograms of compounds; ^1^H-NMR, ^13^C-NMR, COSY, HMBC, HSQC, LC-HRMS, LC-MS, and UV spectra of the new compound, methylenebisastragalin; LC-MS analyses of extracts used for isolation and freshly prepared extracts without heat treatment; and comparative HPLC analyses.
