# Supplementary material for: Urease Inhibitory Activity of Tilia rubra subsp. caucasica V.Engl.: Activity-Guided Fractionation of a Folk Medicine
Source: Molecules. 2026 Nov 1;31(21):3934. doi: 10.3390/molecules31213934 (PMC13764806; doi:10.3390/molecules31213934)
Supplement: Supplementary file 1 [file molecules-31-03934-s001.zip › molecules-4604999-supplementary.pdf]

Supplementary Information

Urease inhibitory activity of *Tilia rubra* subsp. *caucasica* V.Engl.: activity-guided fractionation of a folk medicine

Elif DİLMAÇ[1], İlhan GÜRBÜZ[1], Gökçen EREN[2], Erdal BEDİR[3,*]

[1] Gazi University, Faculty of Pharmacy, Department of Pharmacognosy, Ankara, Türkiye

[2] Gazi University, Faculty of Pharmacy, Department of Pharmaceutical Chemistry, Ankara, Türkiye

[3] İzmir Institute of Technology, Department of Bioengineering, İzmir, Türkiye

E-mail addresses: elifdilmac@gazi.edu.tr (Elif DİLMAÇ), igurbuz@gazi.edu.tr (İlhan GÜRBÜZ), gokcene@gazi.edu.tr (Gökçen EREN), erdalbedir@iyte.edu.tr (Erdal BEDİR)

*Corresponding author.

Contents	Page
Figure S1 HPLC chromatogram of Guaijaverin	3
Figure S2 HPLC chromatogram of trans-Tiliroside	3
Figure S3 HPLC chromatogram of Isoquercitrin	3
Figure S4 HPLC chromatogram of the isolated protocatechuic acid, (b) HPLC chromatogram of the isolated protocatechuic acid spiked with the protocatechuic acid standard using the standard addition method	4
Figure S5 HPLC chromatogram of Methylenebisastragalin	4
Figure S6 ^{1}H-NMR spectrum of Methylenebisastragalin	5
Figure S7 Processed ^{1}H-NMR spectrum of methylenebisastragalin	5
Figure S8 ^{13}C-NMR spectrum of Methylenebisastragalin	6
Figure S9 Processed ^{13}C-NMR spectrum of Methylenebisastragalin	6
Figure S10 COSY spectrum of Methylenebisastragalin	7
Figure S11 Expanded COSY spectrum of methylenebisastragalin	7
Figure S12 Annotated COSY spectrum of methylenebisastragalin	8
Figure S13 Expanded and annotated COSY spectrum of methylenebisastragalin, region I	8
Figure S14 Expanded and annotated COSY spectrum of methylenebisastragalin, region II	9
Figure S15 HMBC spectrum of Methylenebisastragalin	9
Figure S16 Annotated HMBC spectrum of methylenebisastragalin	10
Figure S17 Expanded and annotated HMBC spectrum of methylenebisastragalin	10
Figure S18 Expanded HMBC spectrum of methylenebisastragalin	11
Figure S19 HSQC spectrum of Methylenebisastragalin	11
Figure S20 Annotated HSQC spectrum of methylenebisastragalin	12
Figure S21 Expanded HSQC spectrum of methylenebisastragalin	12
Figure S22 LC-HRMS spectrum of Methylenebisastragalin	13
Figure S23 UV spectrum of Methylenebisastragalin	13
Figure S24 LC-MS spectrum of Methylenebisastragalin	14
Figure S25 LC-MS spectrum of the TRC 80% ethanol extract used for isolation.	14
Figure S26 LC-MS spectrum of the 80% ethanol extract of TRC prepared directly from the plant material without heat treatment	14
Figure S27 LC-MS spectrum of the TRC ethyl acetate subextract used for isolation.	14
Figure S28 LC-MS spectrum of the ethyl acetate extract of TRC prepared directly from the plant material without heat treatment	15
Figure S29 HPLC chromatograms of (a) isolated methylenebisastragalin, (b) the EtOAc extract prepared directly from the plant material without heat treatment, and (c) the overlaid chromatograms of methylenebisastragalin and the EtOAc extract.	15

Figure S1 HPLC chromatogram of Guaijaverin (250 nm)*

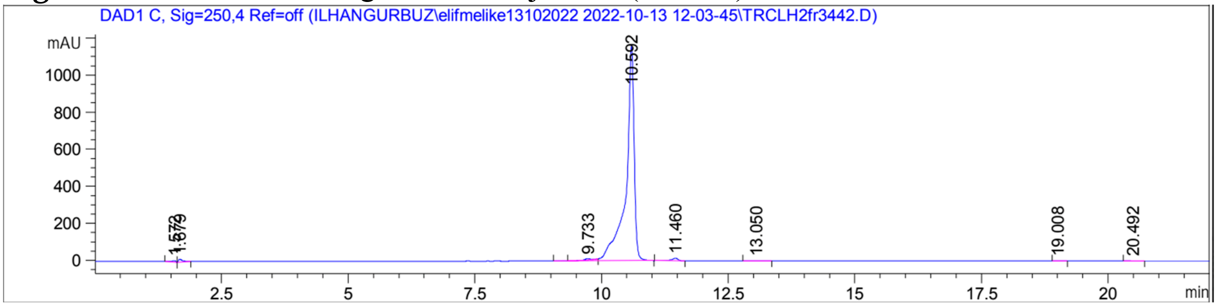

Figure S2 HPLC chromatogram of *trans*-Tiliroside (250 nm)*

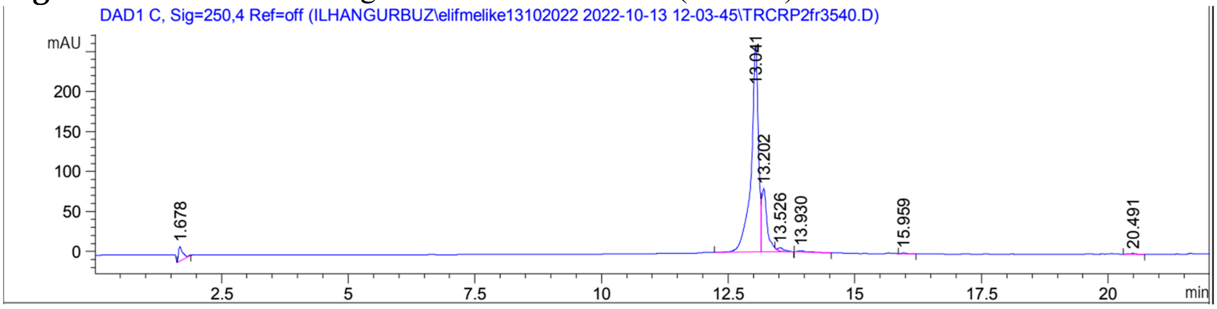

Figure S3 HPLC chromatogram of Isoquercitrin (250 nm)*

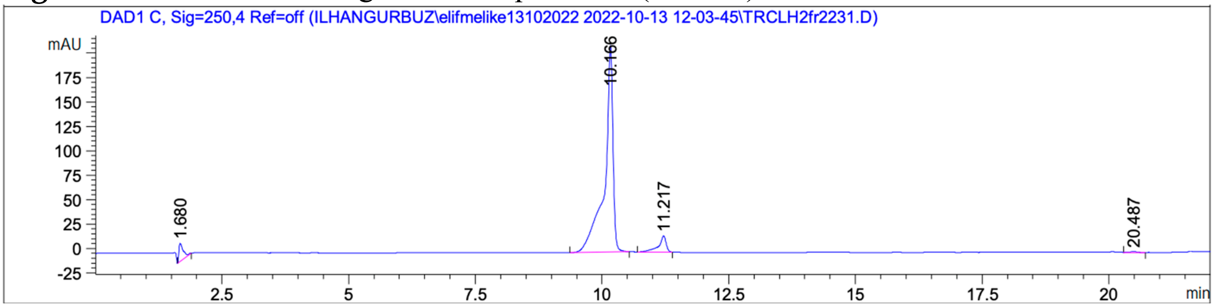

Figure S4 (a) HPLC chromatogram of the isolated protocatechuic acid (250 nm), (b) HPLC chromatogram of the isolated protocatechuic acid spiked with the protocatechuic acid standard using the standard addition method (250 nm)*

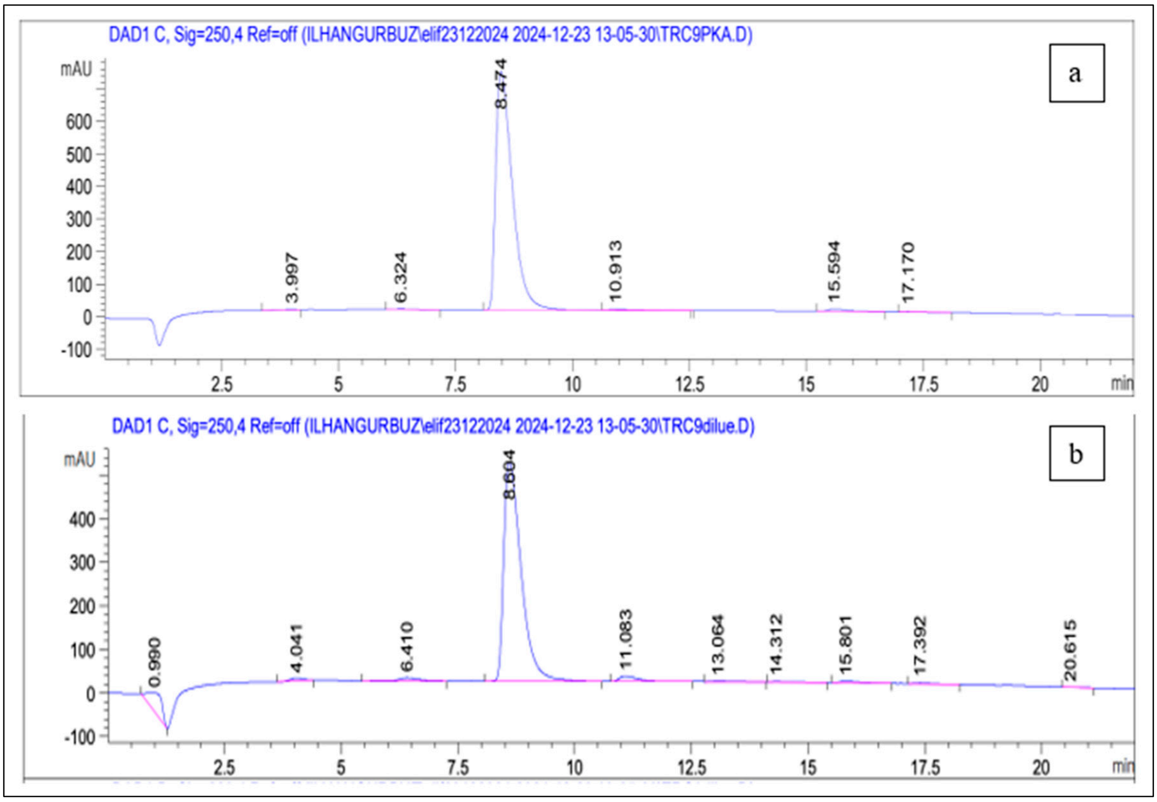

Figure S5 HPLC chromatogram of Isoquercitrin*

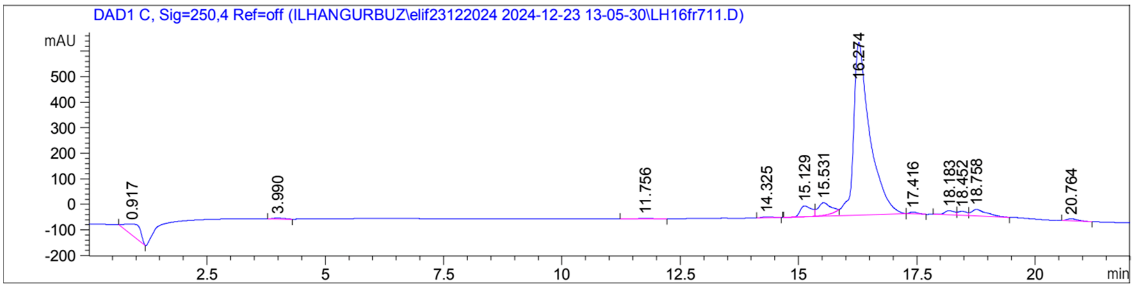

***HPLC conditions:** HPLC analyses were performed using an Agilent 1260 LC system equipped with an ACE 5 C18 column (5 µm, 150 × 4.6 mm) and UV detection (210–365 nm). The column temperature and flow rate were maintained at 25 °C and 1.0 mL/min, respectively. A methanol–water gradient from 20:80 to 100:0 (v/v) over 20 min was applied, followed by 100% methanol for 2 min (total run time: 22 min).

Figure S6 ^{1}H-NMR spectrum of Methylenebisastragalin (CH_3OH-d_4, 500 MHz)

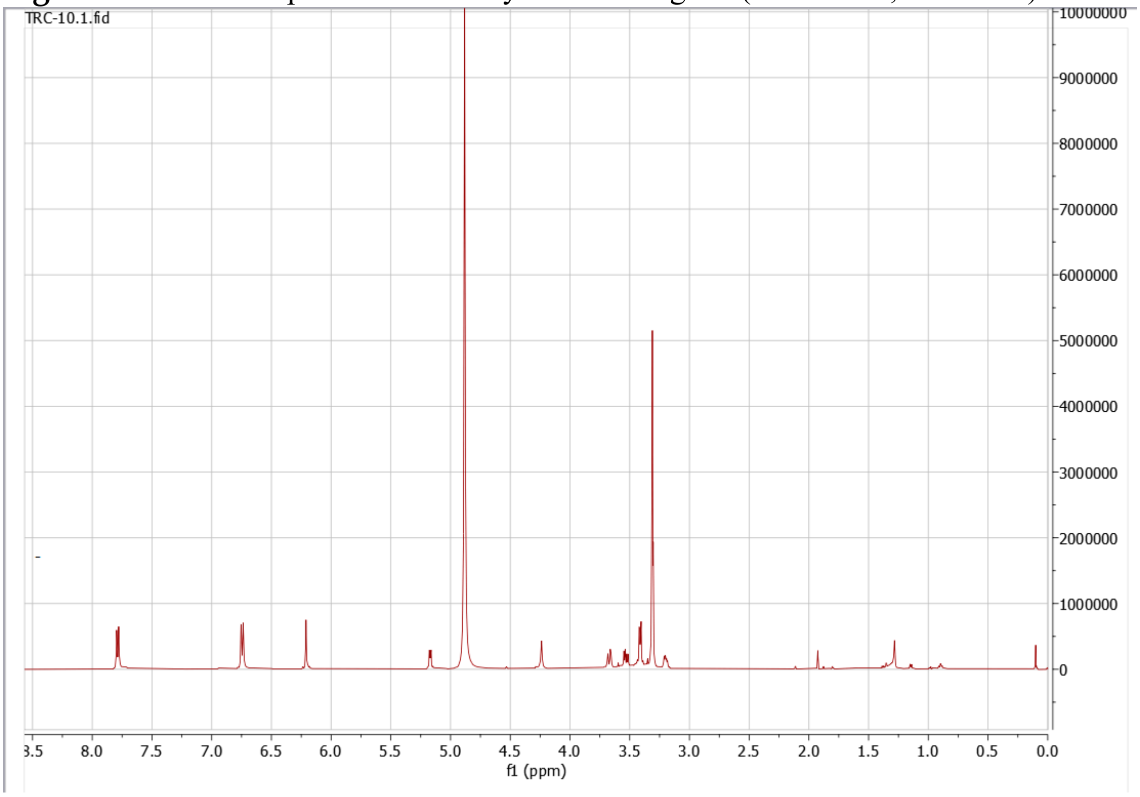

Figure S7 Processed ^{1}H-NMR spectrum of methylenebisastragalin (CD_3OD, 500 MHz)

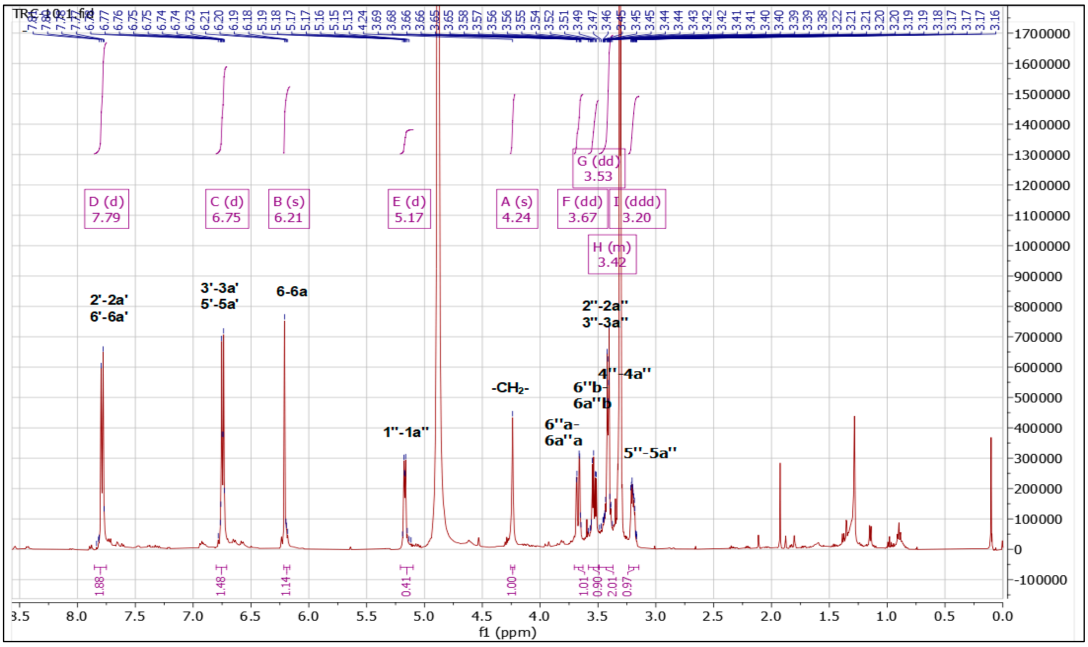

Figure S8 ^{13}C-NMR spectrum of Methylenebisastragalin (CH_3OH-d_4, 125 MHz)

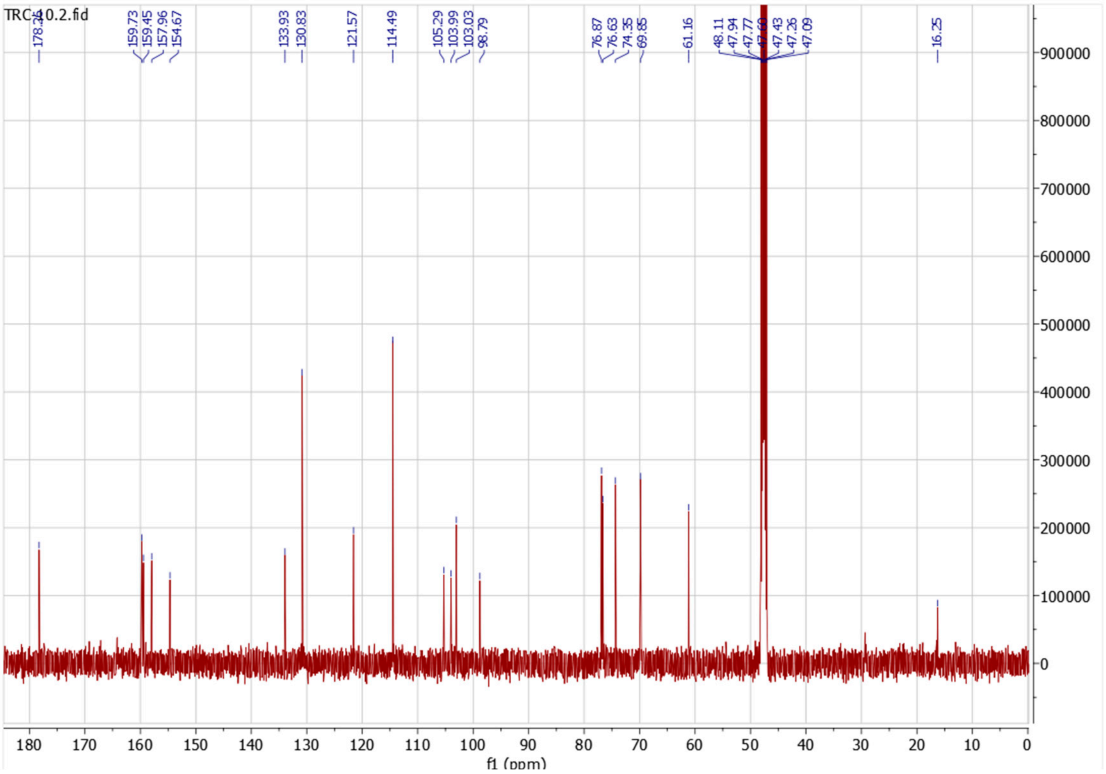

Figure S9 Processed ^{13}C-NMR spectrum of Methylenebisastragalin (CH_3OH-d_4, 125 MHz)

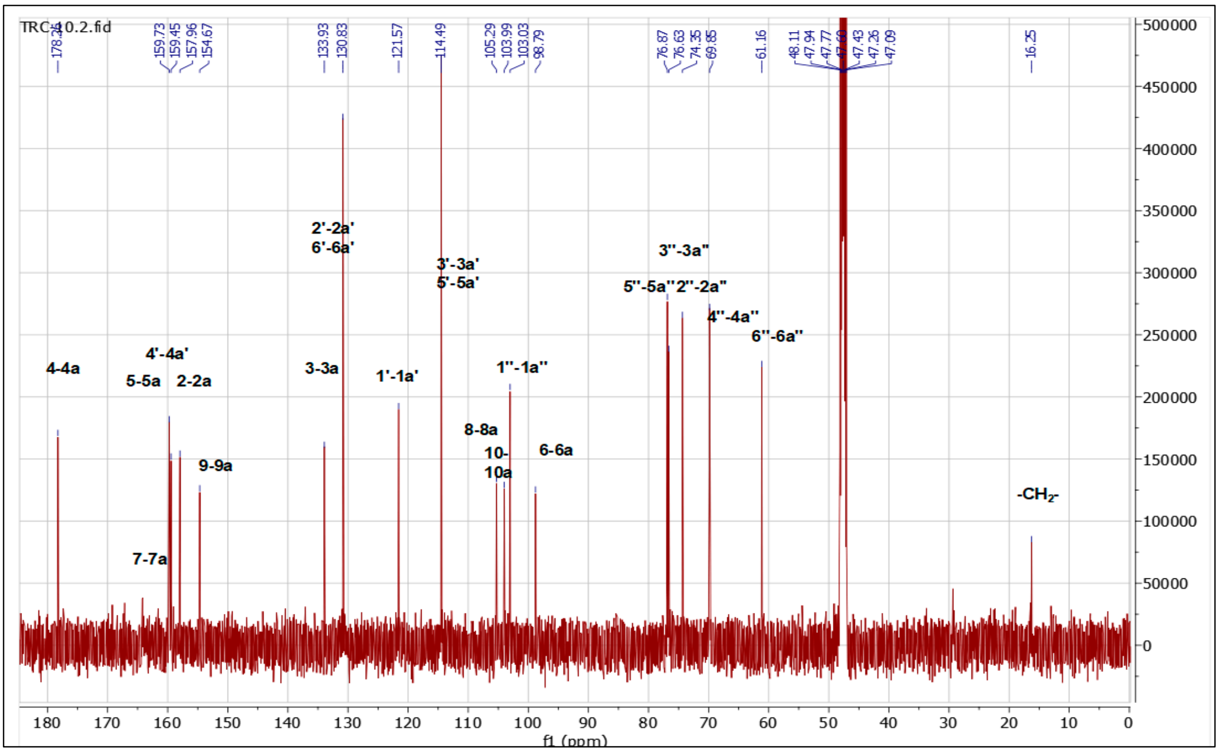

Figure S10 COSY spectrum of Methylenebisastragalin (CH_3OH-d_4, 500 MHz)

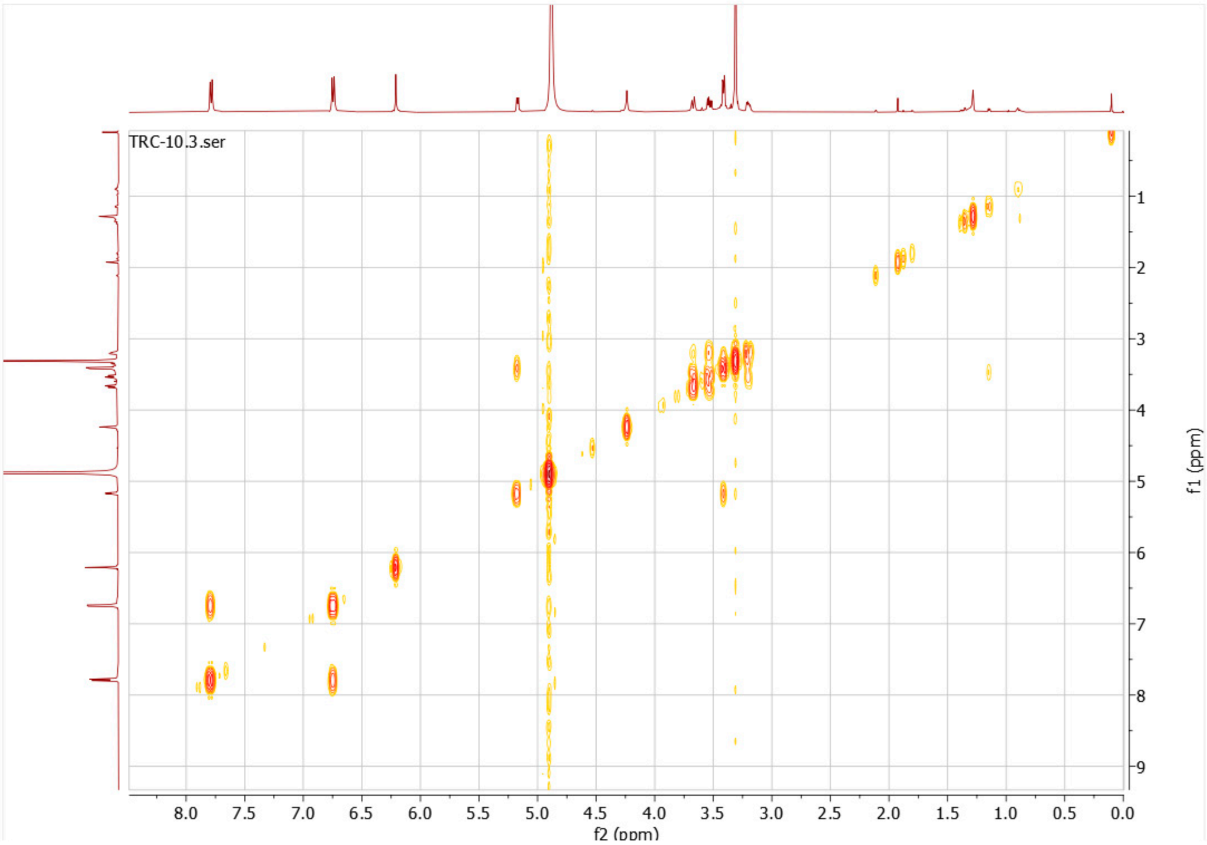

Figure S11. Expanded COSY spectrum of methylenebisastragalin (CD_3OD)

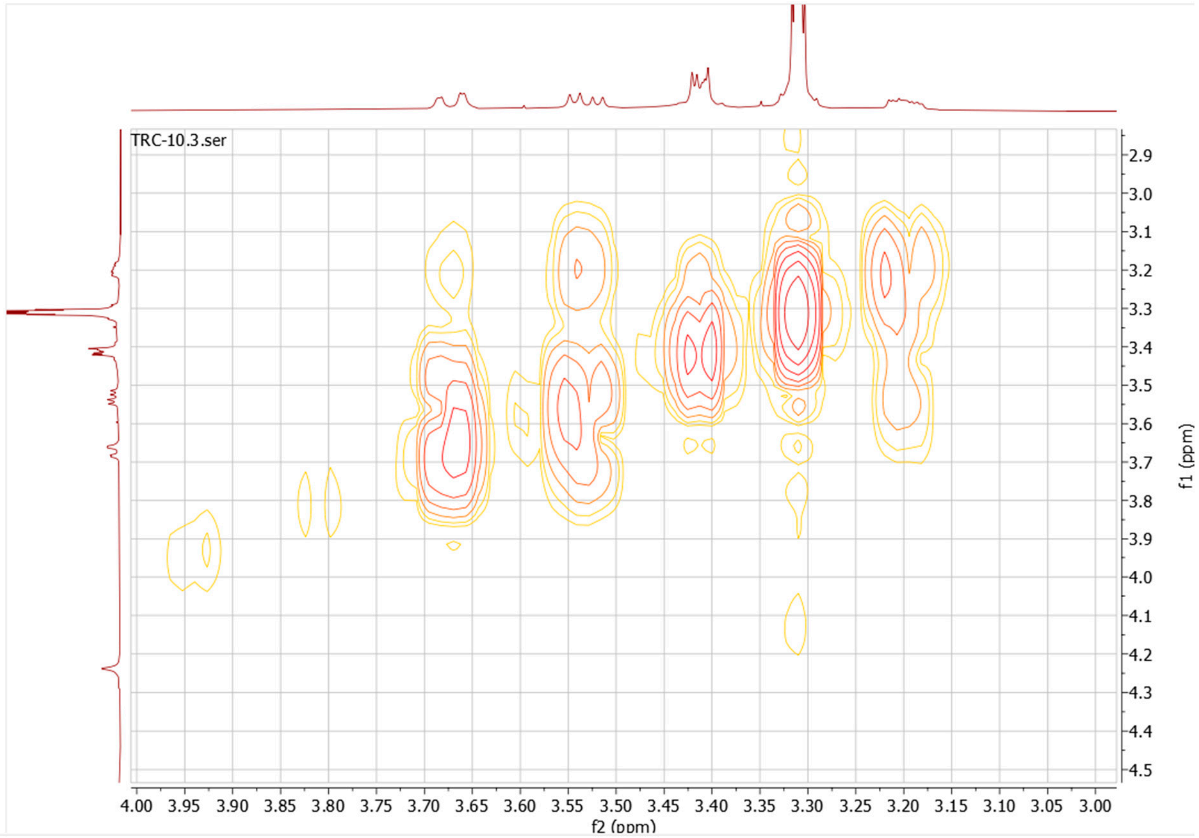

Figure S12. Annotated COSY spectrum of methylenebisastragalin (CD_3OD)

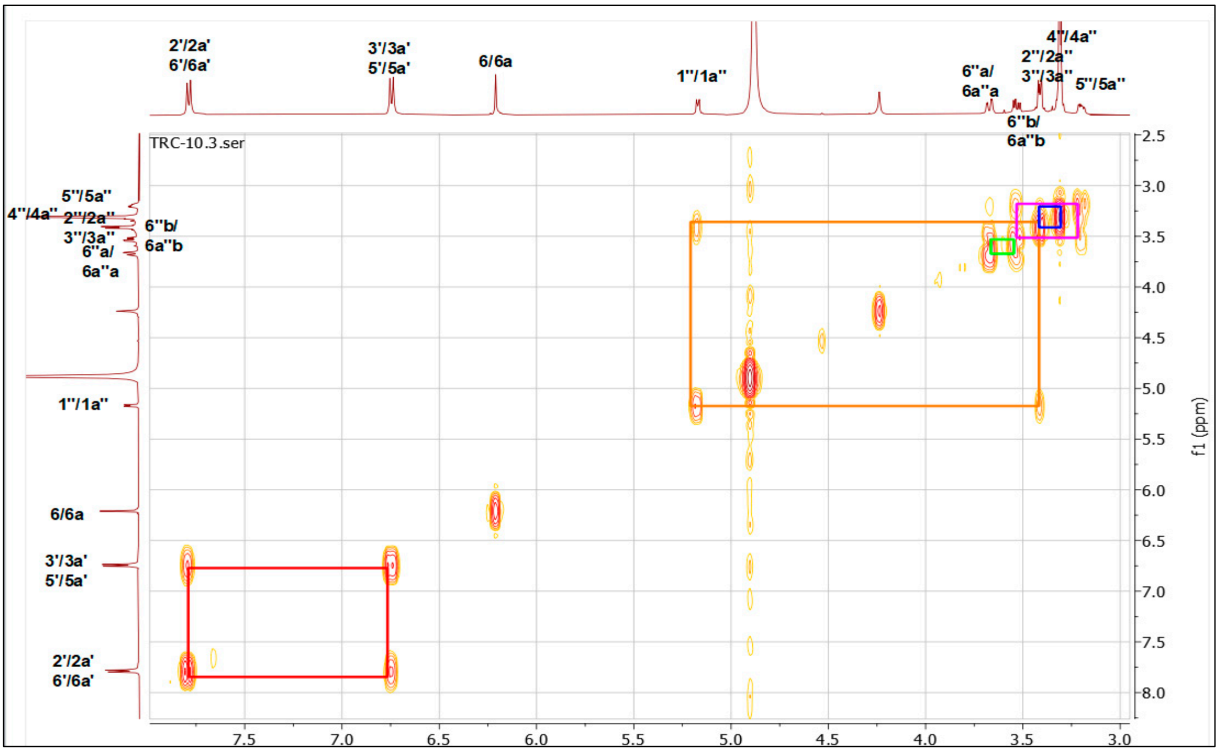

Figure S13 Expanded and annotated COSY spectrum of methylenebisastragalin, region I (CD_3OD)

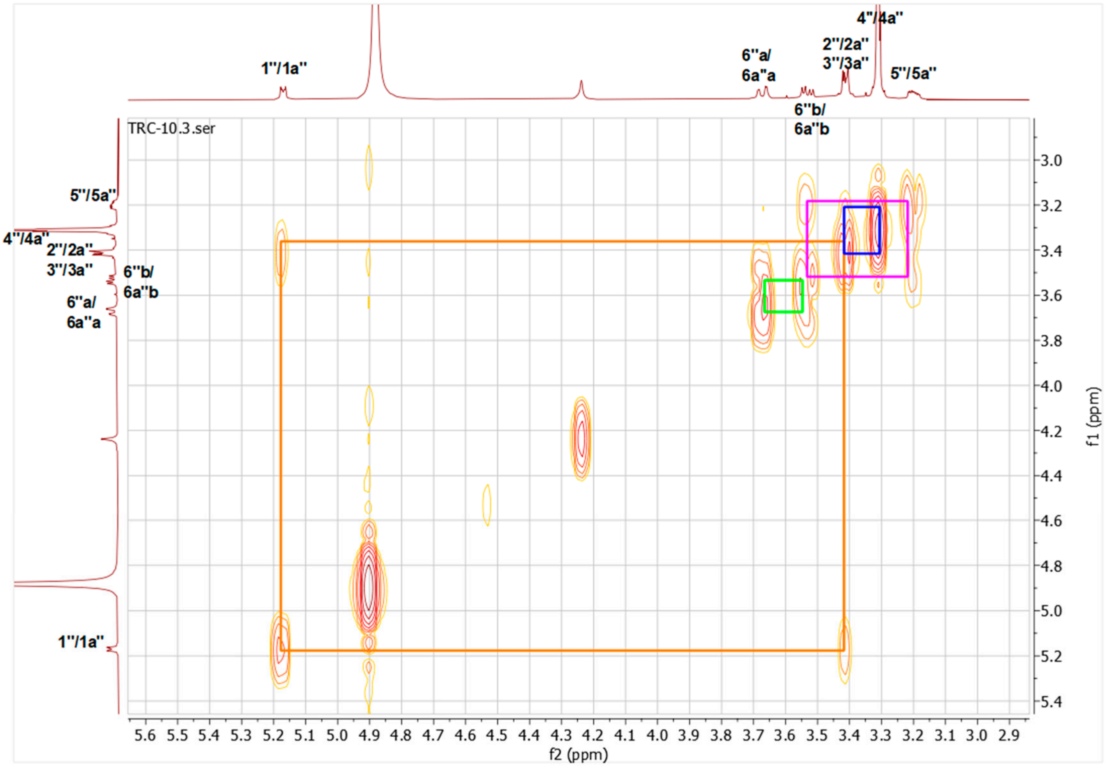

Figure S14 Expanded and annotated COSY spectrum of methylenebisastragalin, region II (CD_3OD)

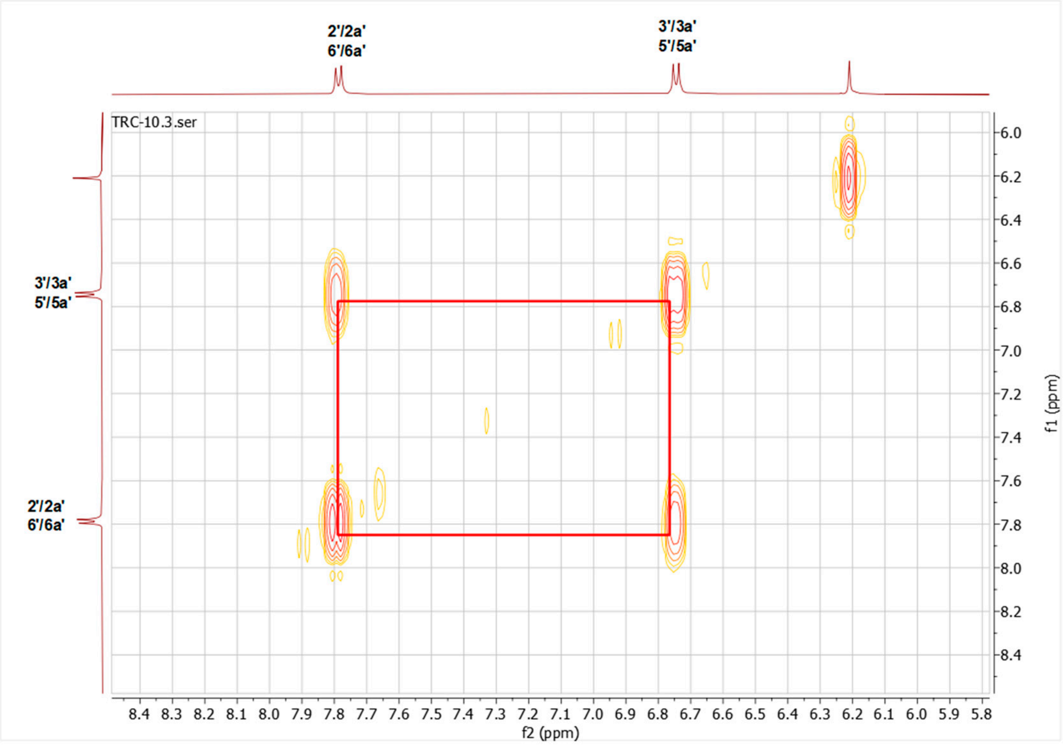

Figure S15 HMBC spectrum of Methylenebisastragalin (CH_3OH-d_4, 500 MHz)

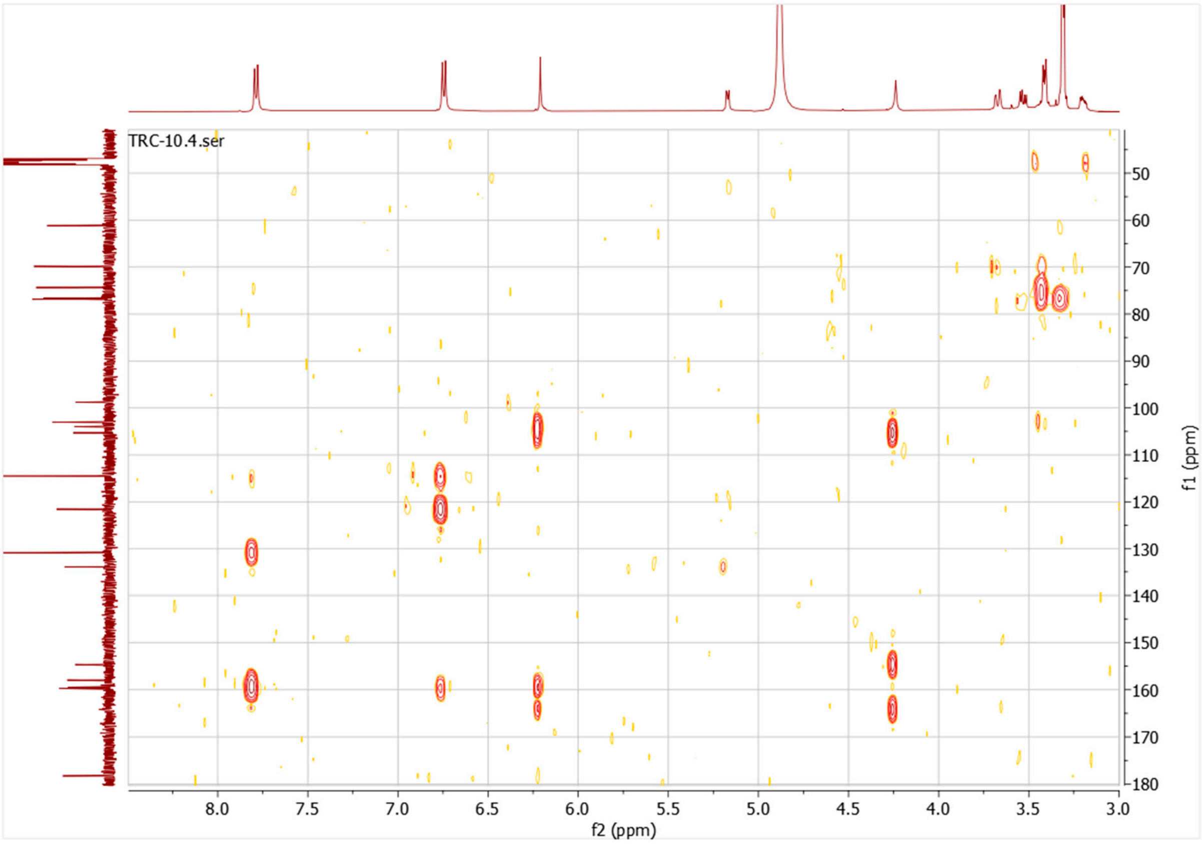

Figure S16 Annotated HMBC spectrum of methylenebisastragalin (CD_3OD)

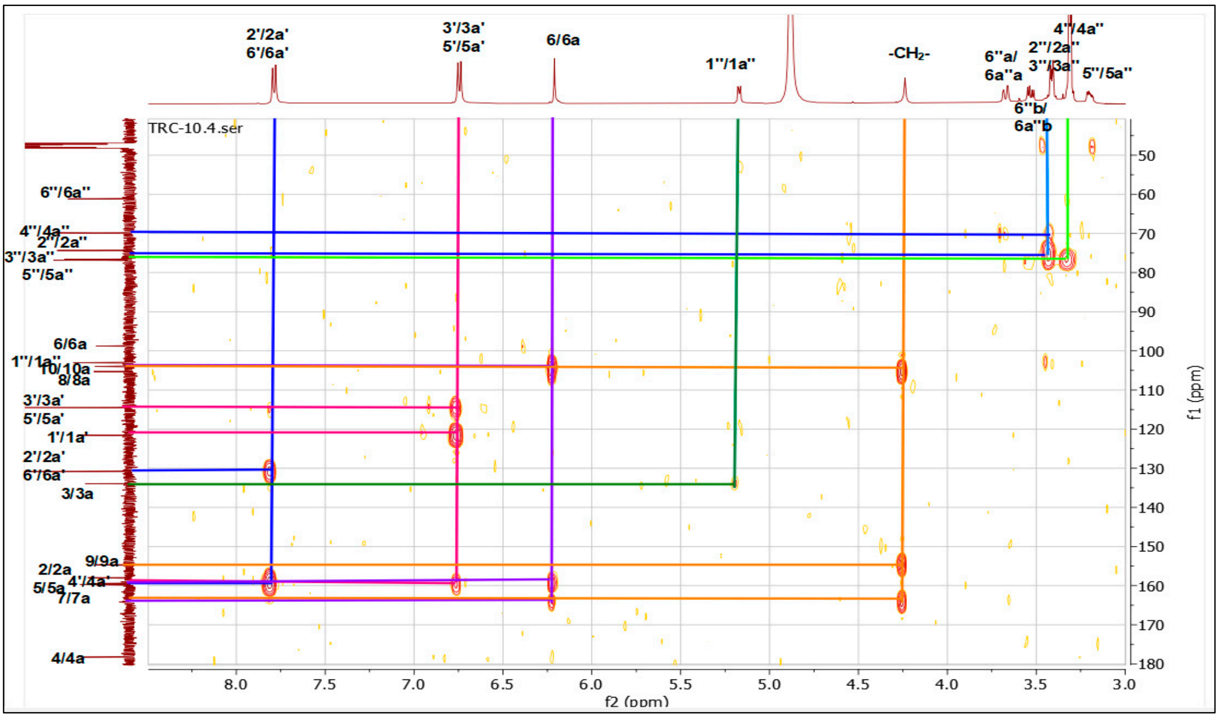

Figure S17 Expanded and annotated HMBC spectrum of methylenebisastragalin (CD_3OD)

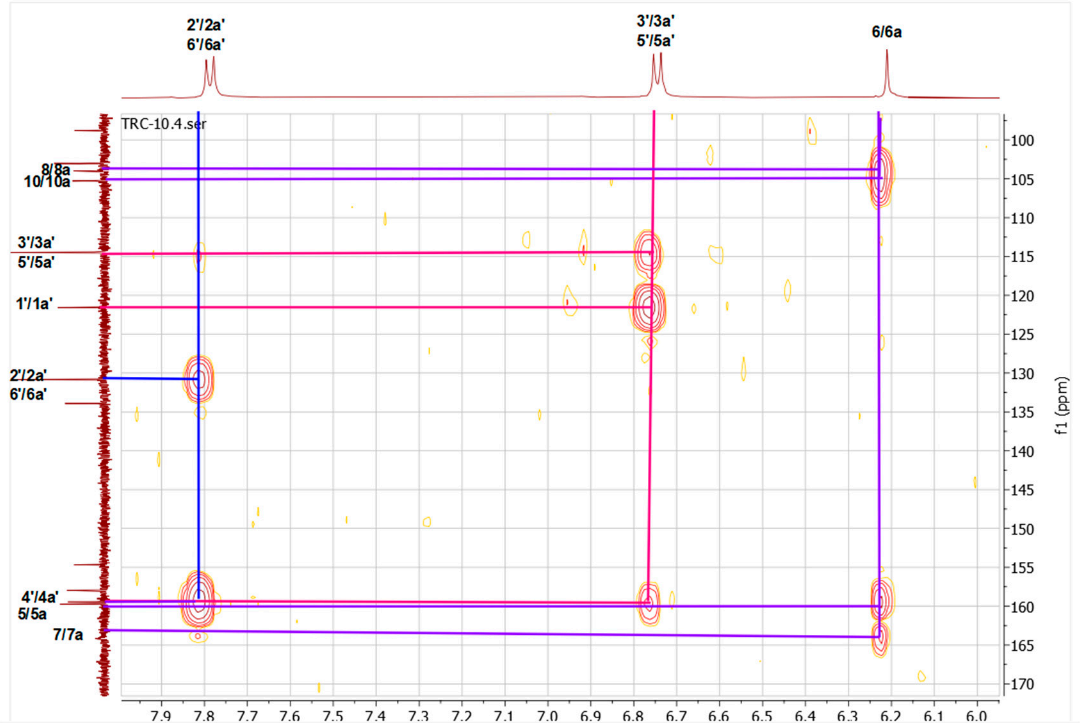

Figure S18 Expanded HMBC spectrum of methylenebisastragalin (CD_3OD)

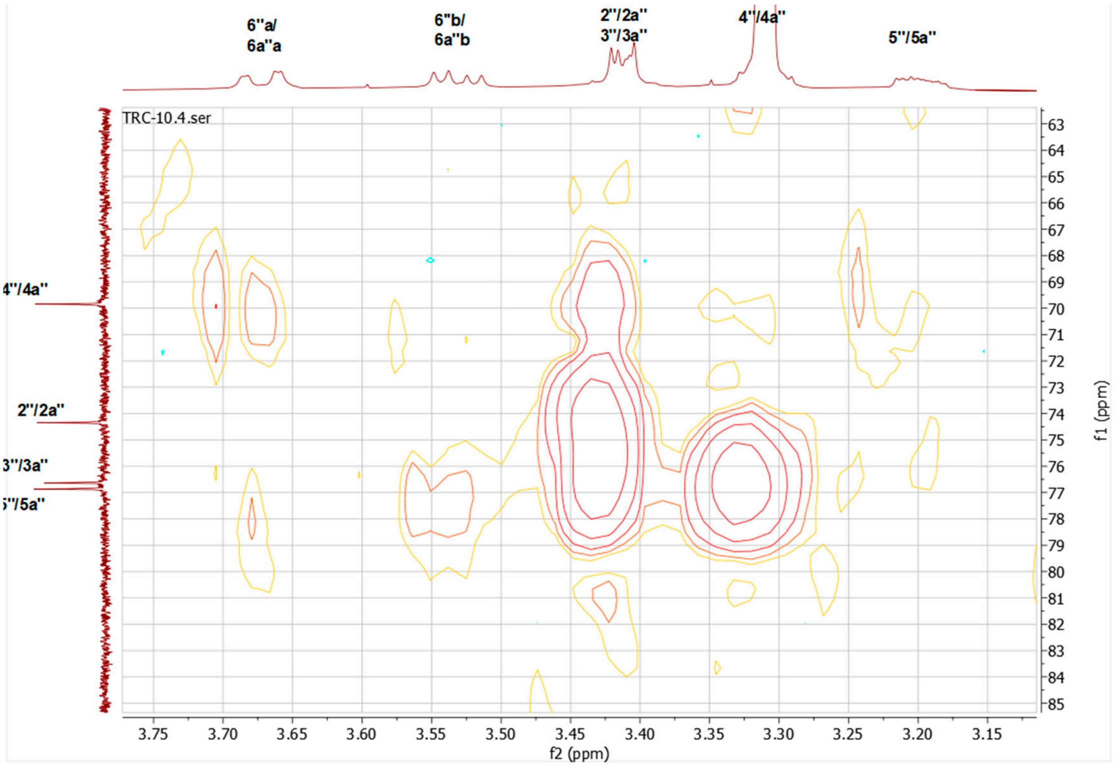

Figure S19 HSQC spectrum of Methylenebisastragalin (CH_3OH-d_4, 500 MHz)

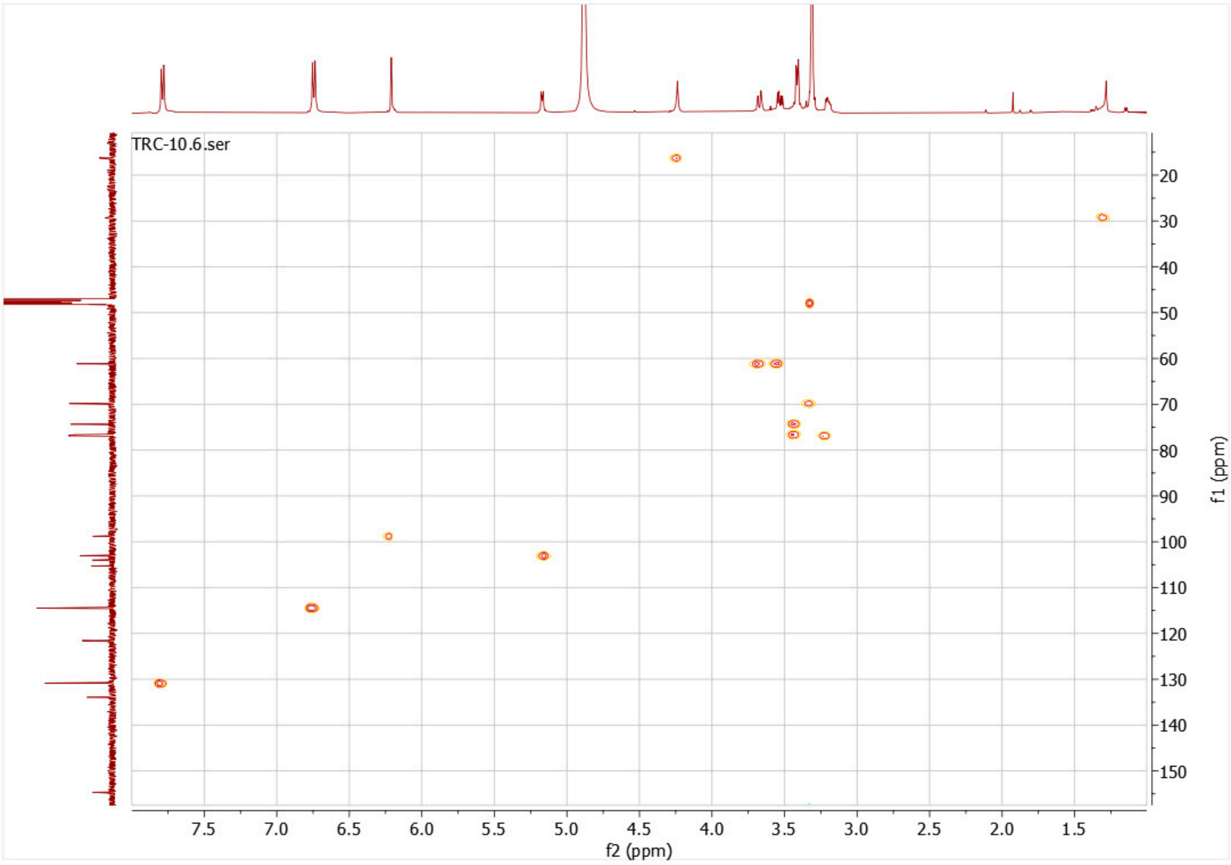

Figure S20 Annotated HSQC spectrum of methylenebisastragalin (CD_3OD)

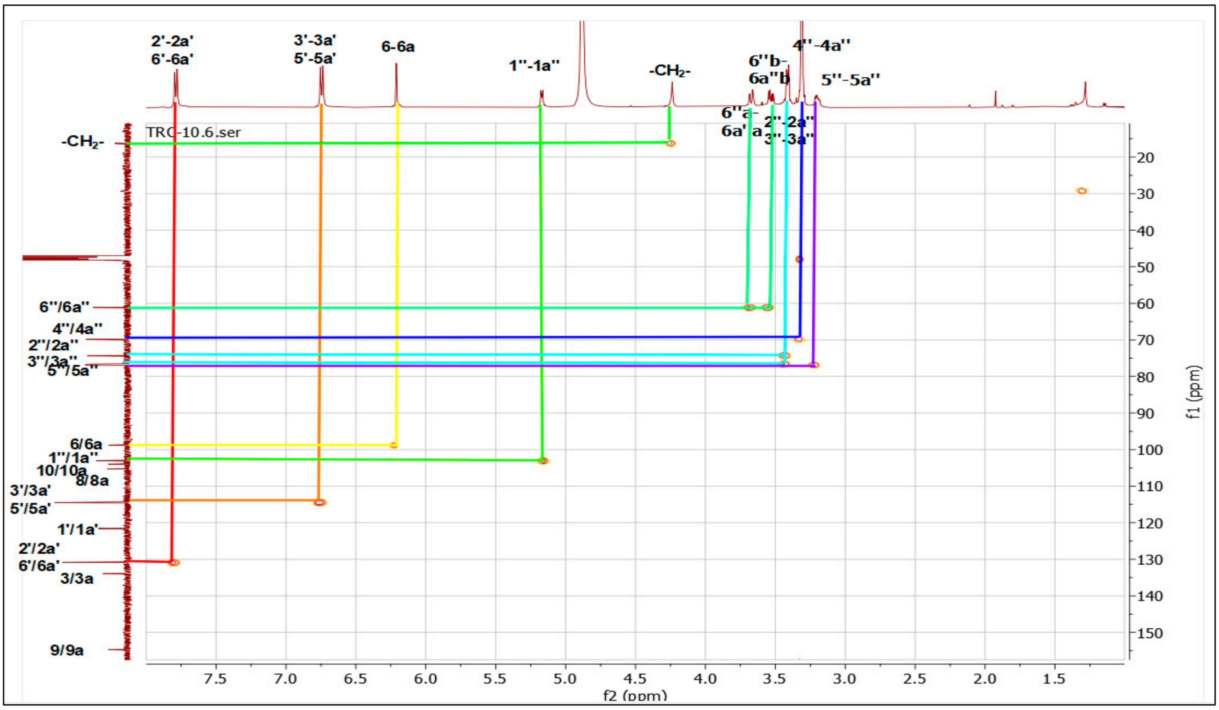

Figure S21 Expanded HSQC spectrum of methylenebisastragalin (CD_3OD)

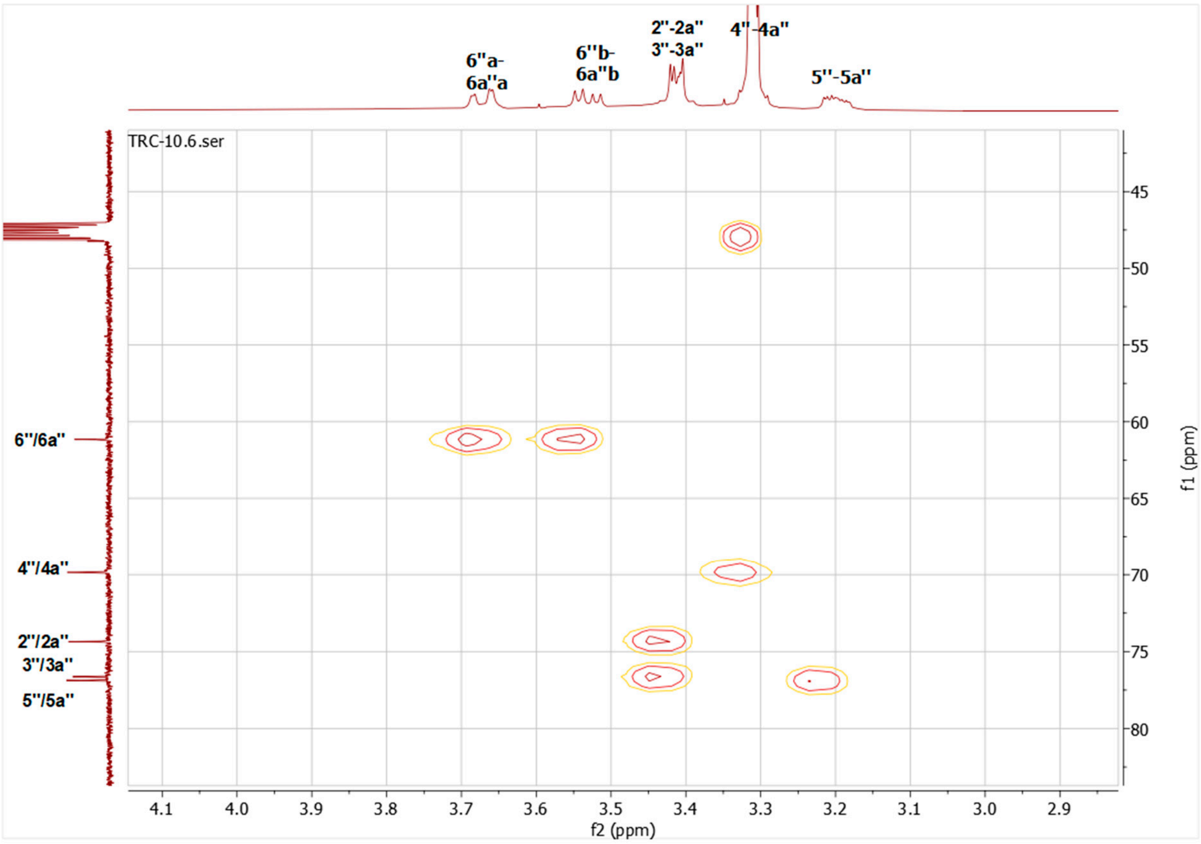

Figure S22 LC-HRMS spectrum of Methylenebisastragalin

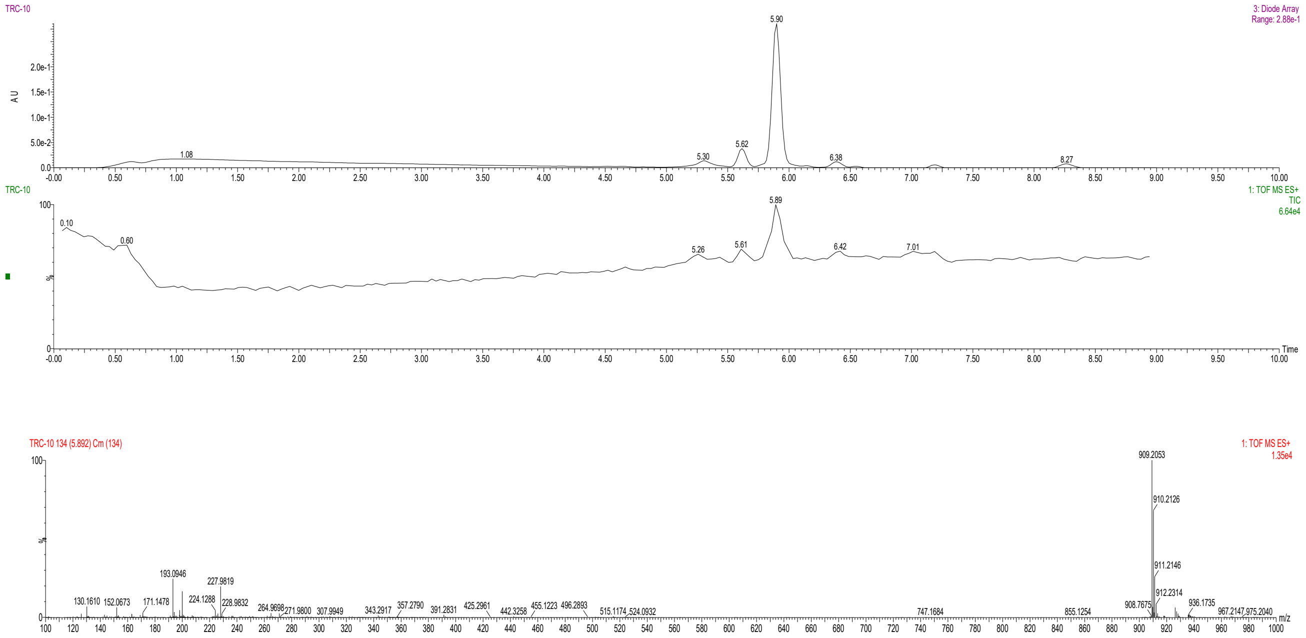

Figure S23 UV spectrum of Methylenebisastragalin

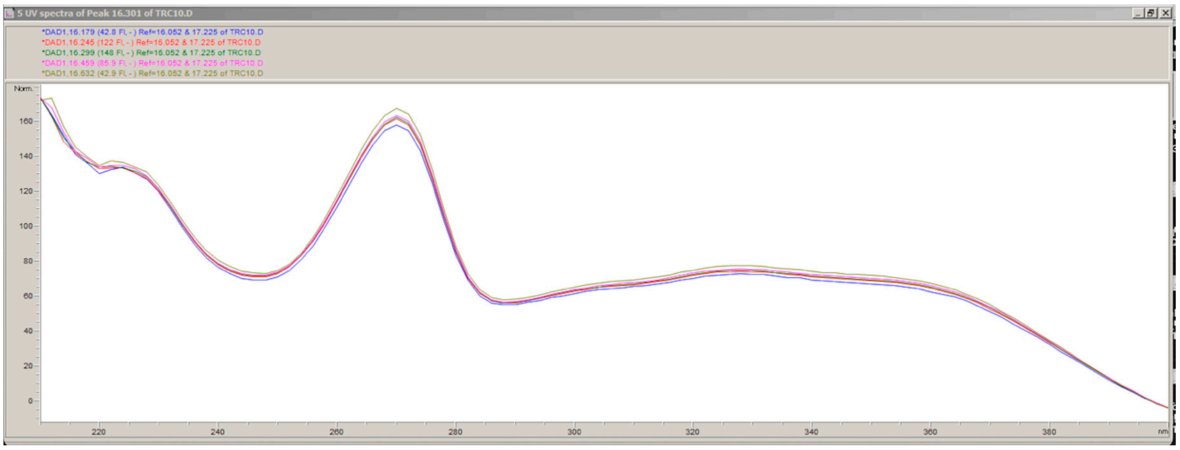

Figure S24 LC-MS spectrum of Methylenebisastragalin**

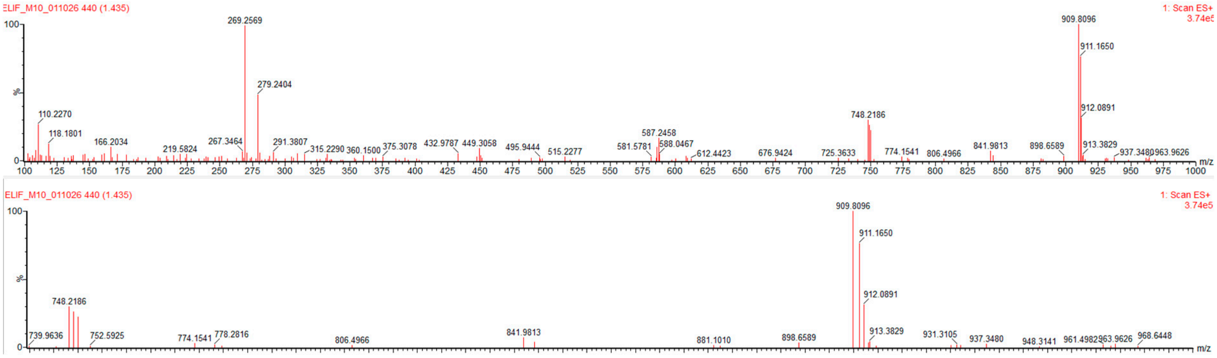

Figure S25 LC-MS spectrum of the TRC 80% ethanol extract used for isolation**

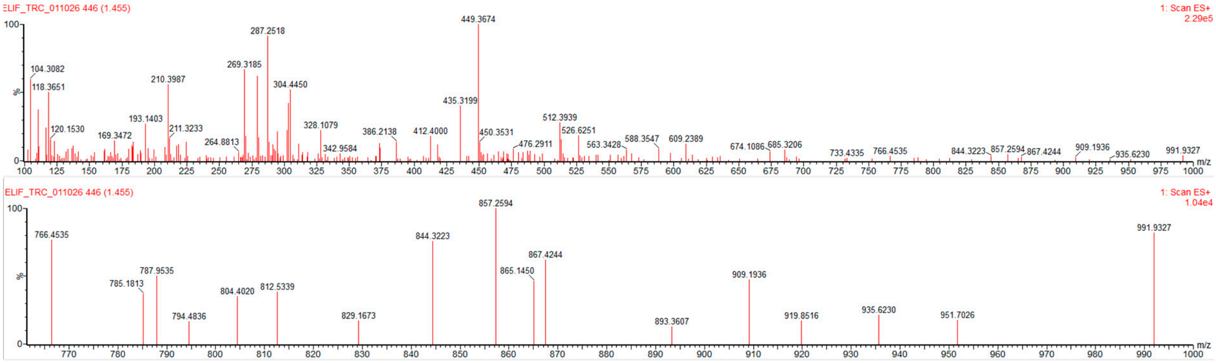

Figure S26 LC-MS spectrum of the 80% ethanol extract of TRC prepared directly from the plant material without heat treatment**

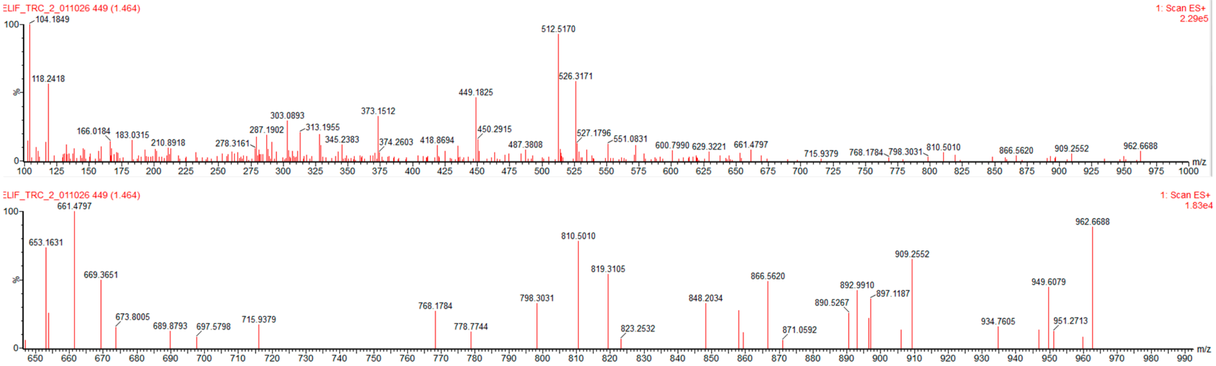

Figure S27 LC-MS spectrum of the TRC ethyl acetate subextract used for isolation**

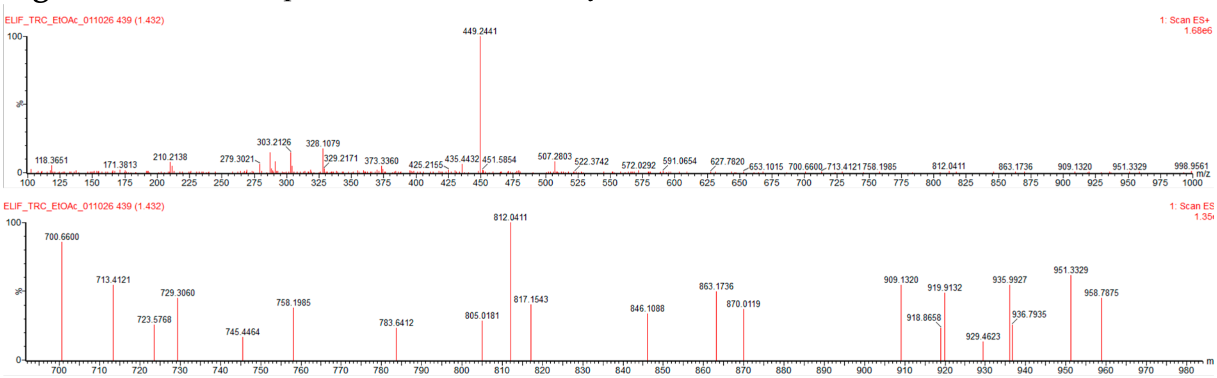

Figure S28 LC-MS spectrum of the ethyl acetate extract of TRC prepared directly from the plant material without heat treatment**

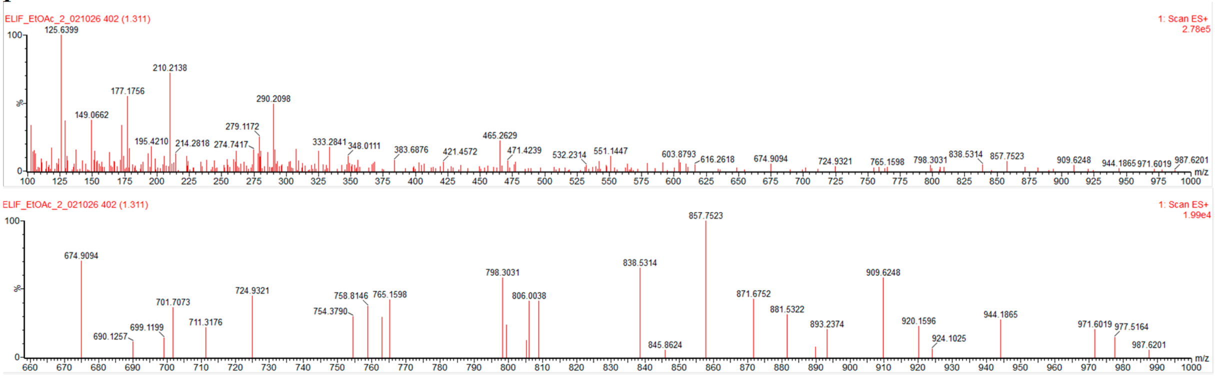

** The mass spectra were acquired using a Waters ACQUITY QDa II Mass Detector.

Figure S29. HPLC chromatograms of (a) isolated methylenebisastragalin, (b) the EtOAc extract prepared directly from the plant material without heat treatment, and (c) the overlaid chromatograms of methylenebisastragalin and the EtOAc extract (250 nm).

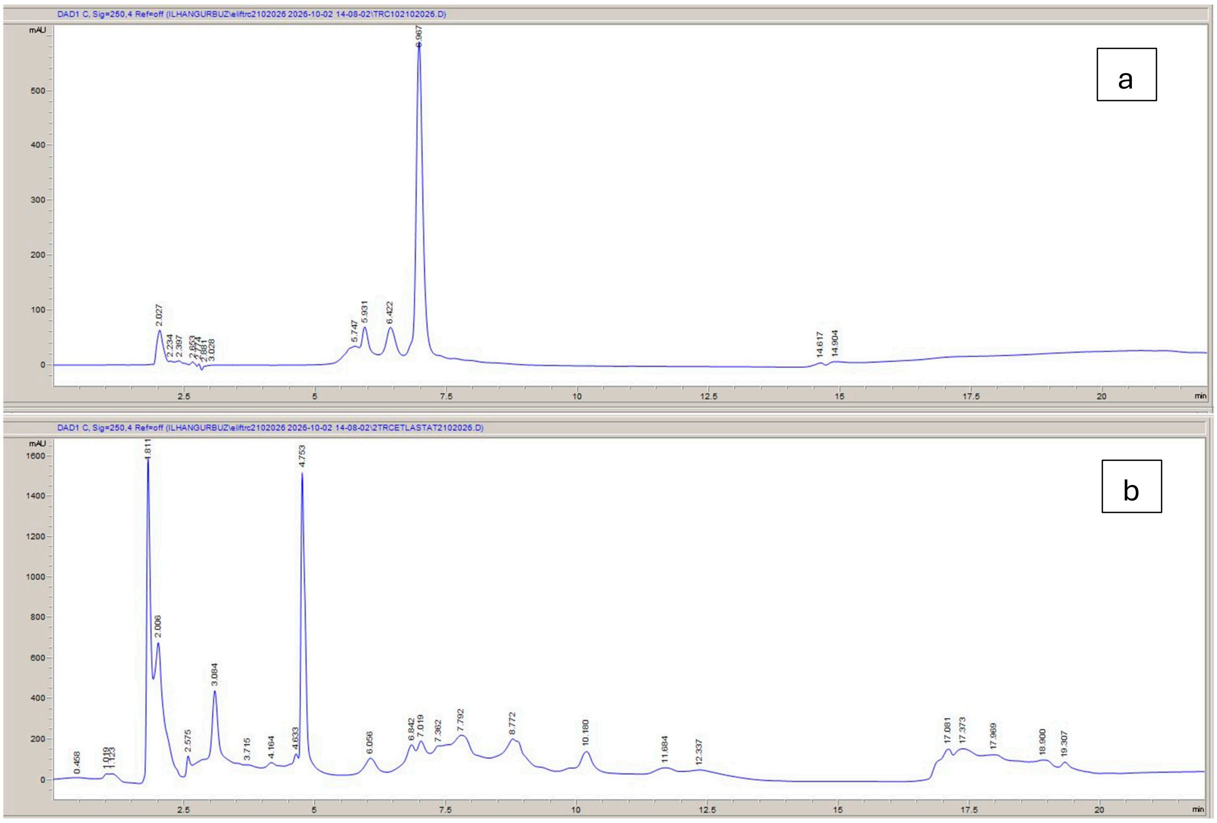

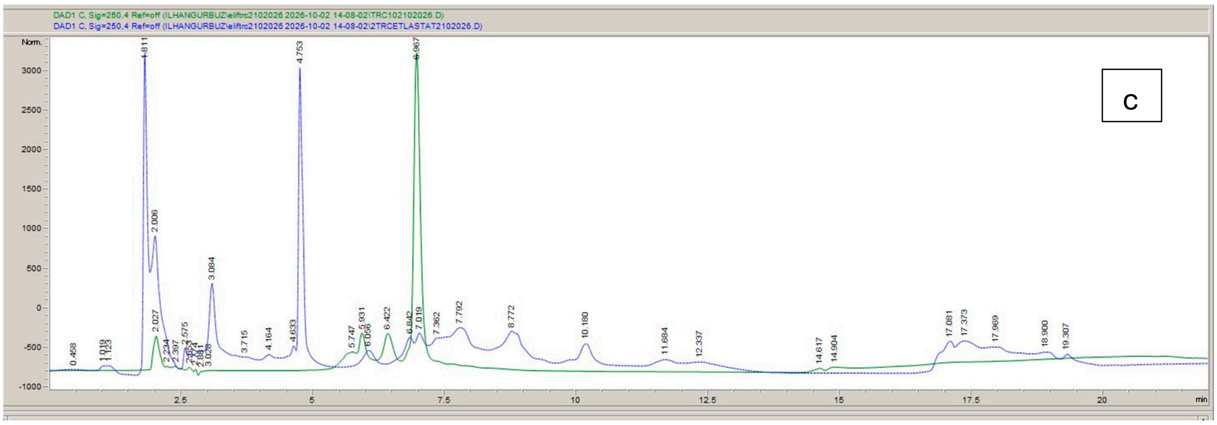
DAD1 C, Sig=250,4 Ref=off (ILHANGURBUZ\eliftrc2102026 2026-10-02 14-08-02\TRC102102026.D)
DAD1 C, Sig=250,4 Ref=off (ILHANGURBUZ\eliftrc2102026 2026-10-02 14-08-02\2TRCETLASTAT2102026.D)
Norm.
3000
2500
2000
1500
1000
500
0
-500
-1000
2.5
5
7.5
10
12.5
15
17.5
20
min
c
